# Psychology of Eating the Future: Consumer Acceptance, Digital Influence and Behavioral Drivers of Novel Foods

**DOI:** 10.3390/foods15142471

**Published:** 2026-07-12

**Authors:** Muhammad Faisal Manzoor, Muhammad Talha Afraz, Muhammad Waseem, Zahoor Ahmed

**Affiliations:** 1 Guangdong Provincial Key Laboratory of Intelligent Food Manufacturing, School of Food Science and Engineering, Foshan University, Foshan 528225, China; faisaluos26@gmail.com; 2 School of Food Science and Engineering, South China University of Technology, Guangzhou 510641, China; m.talha.afraz@gmail.com; 3 Department of Food Science and Technology, Faculty of Agriculture and Environment, The Islamia University of Bahawalpur, Bahawalpur 63100, Pakistan; muhammadwaseem9499@gmail.com; 4 Human Nutrition and Dietetics School of Food and Agricultural Sciences, University of Management Technology, Lahore 54770, Pakistan

**Keywords:** novel food, consumer behavior, alternative proteins, food neophobia, sustainability, digital marketing

## Abstract

The accelerating urgency of global public health challenges, biodiversity loss, and climate change has driven rapid innovation in novel foods and alternative proteins, including cultured cells, fermentation-derived components, plant-based meats, insects, and algae, which promise nutritious, sustainable, and ethical dietary choices with lower environmental footprints. Although technologies have advanced, consumer perception and preferences remain key hindrances due to perceptual, cultural, and sensory challenges. This semi-systematic narrative literature review aims to incorporate interdisciplinary studies (2020–2025) that span sensory science, AI-driven marketing, behavioral economics, and policy analysis to explore consumer incentives, barriers, and intervention approaches associated with novel food categories. Of 1260 initial records, 310 duplicates were removed, 530 were excluded at title/abstract screening, 233 were excluded at full-text review, leaving 197 studies for the final synthesis. The focus is on understanding cultural contexts, cognitive biases, digital and social influences, and the global framing impacts that shape consumer adoption. Consumer perceptions and preferences are primarily influenced by health benefits, ethical concerns, and environmental sustainability; however, neophobia, sensory unfamiliarity, trust deficits, and price temper these factors. Preliminary evidence suggests that AI-generated personalization, transparent labeling, behavioral nudges, and social norms may be useful tools for overcoming resistance to change, though the effectiveness of AI-driven personalization in actual purchasing behavior is not yet firmly established. Cultural diversity affects acceptance routes, with culturally established insect consumption differing from Western neophobia. Future studies should integrate interdisciplinary methodologies, longitudinal cross-cultural analyses, and innovative technologies to enhance communication and product design.

## 1. Introduction

**Figure 1:**
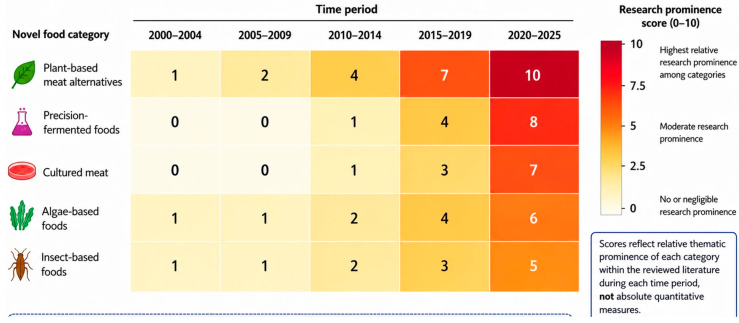
Research activity and thematic prominence of major novel food categories over time. Conceptual heatmap illustrating the relative research prominence of major novel food categories, including plant-based meat alternatives, precision-fermented foods, cultured meat, algae-based foods, and insect-based foods, across the periods. Scores range from 0 (no or negligible research prominence) to 10 (the highest relative research prominence) and represent the relative thematic prominence of each category within the reviewed literature. The figure is intended to provide a conceptual comparison of research activity over time rather than a quantitative or market-based analysis.

Globally, the food system faces exceptional pressure to evolve in response to rising climate concerns and mounting public health issues. Traditional animal agriculture is responsible for biodiversity loss, land degradation, and substantial greenhouse gas emissions, all of which are highly acknowledged as unsustainable [1]. The increasing burden of diet-related illnesses underscores the need for nutrient-rich, healthier choices [2]. These two factors have driven innovation in novel foods and alternative proteins, including those containing cultured cellular products, algae, plant-based meats, fermentation-derived components, and insects [3]. By leveraging advanced biotechnologies and underutilized biological resources, novel foods promise reduced environmental footprints while meeting diverse nutritional needs [4]. Figure 1, a qualitative trend chart, illustrates the relative evolution in research focus and consumer interest for the five novel food categories from 2020 to 2025. Intensity values (scale of 0–10) represent qualitative estimates of relative publication volume and market attention, synthesized from database searches in Scopus, Web of Science, and Google Scholar, supplemented by industry market reports. These values are indicative and not statistically derived; they are intended to convey directional trends rather than precise quantitative measures.

Despite advancements in novel technologies and extended product portfolios, consumer preference and acceptance remain key barriers to general adoption. Understanding why consumers accept or reject novel foods is a complex process that requires a holistic perspective, integrating food technology, social psychology, behavioral economics, and marketing science [5]. The recent literature highlights that acceptance is based not only on price and sensory attributes but also on factors such as cultural norms, health benefits, technology framing, digital social influences, and ethical considerations. For example, consumers’ beliefs are deeply influenced by how novel foods are displayed, such as using terms like “clean” or “natural” or analogies to conventional approaches (e.g., fermentation) that improve legitimacy [6].

This review aims to compile a body of recent interdisciplinary literature that explains the fundamental drivers and barriers to consumer acceptance and preferences of innovative novel food types, including insect proteins, algae-based products, cellular agriculture, and precision fermentation. Evaluating behavioral economics insights, such as status quo bias and loss aversion, alongside novel data analytical approaches, including social media mining and AI-driven marketing, it highlights evolved approaches for influencing consumer perspectives [7]. Furthermore, it examines how pricing structures, sensory challenges, cultural neophobia, and trust issues shape market routes. This observation highlights the potential of transparent labeling, digital personalization, and tailored nudges to promote sustainable food alternatives at scale [8].

An emerging marketing module leverages AI algorithms and big data to enable real-time adaptation and segmentation based on consumer responses and feedback, thereby optimizing products to align with evolving preferences [9]. Concurrently, virtual technologies, including virtual reality, present novel experiential pathways to improve food familiarity and reduce neophobia [10]. Cross-cultural stances demonstrate diverse patterns of acceptance. While insect consumption is often stigmatized in Western culture, it is widely accepted as a nutrient-rich food source in many parts of the world, offering opportunities for culturally driven market approaches [11].

This review integrates policy frameworks, consumer psychology, and advances in food science to elucidate pathways for bridging the innovation–adoption gap. It endorses a consumer-centric interdisciplinary strategy for integrating novel foods into sustainable dietary systems globally, offering ethical food production, improved general health, and climate mitigation. It should be noted, however, that these benefits are not uniform across all novel food categories or production systems. Ethical production, health outcomes, and climate performance vary substantially across products, production scale, and supply chain context. These claims should therefore be understood as directional tendencies supported by evidence under specific conditions, rather than universal guarantees applicable to all novel foods.

### 1.1. Review Methodology

This study employs a semi-systematic narrative review methodology to synthesize the interdisciplinary literature on consumer acceptance and preferences for novel foods published between January 2020 and August 2025. The decision to focus on these five years was deliberate: it captures the most recent and rapidly evolving phase of consumer research in novel foods, reflecting post-COVID-19 dietary shifts, the commercialization of precision fermentation and cultivated meat, and the proliferation of AI-driven food marketing. Where seminal foundational studies predating 2020 are essential to theoretical grounding (e.g., the theory of planned behavior and food neophobia scales), they are selectively integrated and clearly identified. A comprehensive literature search was conducted across four major academic databases: Scopus, Web of Science, PubMed, and Google Scholar. The primary search strings used were “novel food” OR “alternative protein” OR “plant-based meat” OR “cultured meat” OR “insect-based food” OR “precision fermentation” OR “algae-based food” AND “consumer acceptance” OR “consumer preference” OR “consumer perception” OR “willingness to try” OR “food neophobia”. Additional targeted searches were conducted for individual novel food categories combined with terms such as “sustainability”, “sensory”, “behavioral economics”, “AI marketing”, “digital influence”, and “labeling”. The search was completed in August 2025.

**Figure 2:**
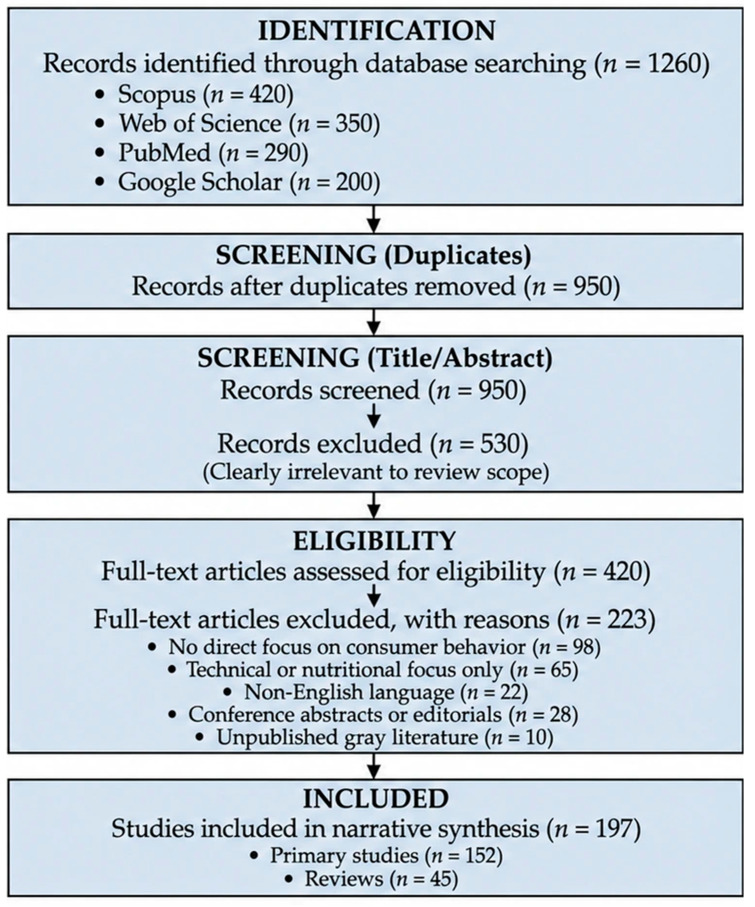
PRISMA 2020 flow diagram of the study selection process.

Articles were included if they: (a) were published in peer-reviewed journals in English; (b) focused on consumer attitudes, perceptions, acceptance, or preferences for one or more novel food categories; (c) were published between January 2020 and August 2025; and (d) provided empirical, review, or theoretical contributions directly relevant to the scope of this review. Articles were excluded if they focused exclusively on the technical production or nutritional composition of novel foods without addressing consumer behavior or if they were conference abstracts, editorials, or unpublished gray literature (e.g., thesis, preprints, and institutional internal reports). However, a selective subset of gray literature was deliberately included, specifically industry market reports and regulatory documents from official governmental or international bodies. These sources were cited only when no peer-reviewed article was available and when they provided essential contextual, policy, or market data critical to the review scope. The selective inclusion is acknowledged as a methodological limitation, and all such sources are clearly identified in the reference list and cited appropriately in the text. Following the initial database search, approximately 1260 records were identified. After removing 310 duplicate records, 950 unique records remained. These were screened by title and abstract against the inclusion criteria, resulting in the exclusion of 530 records that were clearly irrelevant to the review scope. The remaining 420 full-text articles were assessed for eligibility. Of these, 233 were excluded for the following reasons: (a) no direct focus on consumer behavior (n = 98), (b) technical or nutritional focus only (n = 65), (c) non-English language (n = 22), (d) conference abstracts or editorials (n = 28), and (e) unpublished gray literature (n = 10). The remaining 197 primary studies and reviews that met all inclusion criteria form the empirical basis for this narrative synthesis (Figure 2).

### 1.2. Scope and Operational Definitions

To ensure terminological consistency throughout this review, the following working definitions are adopted based on established regulatory and scientific frameworks: “Novel foods” is used as the primary umbrella term, consistent with the European Union’s Novel Food Regulation (EU) 2015/2283, which defines novel foods as foods that were not consumed to a significant degree within the EU before 15 May 1997. This review focuses specifically on five categories that have attracted substantial consumer research attention since 2020: (a) plant-based meat alternatives (PBMAs), including products derived from soy, pea, wheat gluten, and mycoprotein that are formulated to replicate animal meat in sensory profile; (b) cultured meat (also referred to as cell-cultivated or lab-grown meat), produced through the in vitro culture of animal cells; (c) precision fermentation-derived ingredients, produced by genetically engineered microorganisms (bacteria, yeasts, or fungi) to synthesize specific proteins, fats, or bioactive compounds; (d) insect-based proteins, derived from edible insect species (e.g., Acheta domesticus and Tenebrio molitor) approved for human consumption in relevant regulatory jurisdictions; and (e) algae-based foods, encompassing both macroalgae (seaweed) and microalgae (e.g., Spirulina, Chlorella) incorporated as food ingredients or protein sources.

“Alternative proteins” is used as a broader category encompassing all five novel food types above, as well as mycoprotein and fermentation-derived dairy analogs, where referenced in cited studies. Further, “3D-printed foods” and “GM (genetically modified) foods” are discussed, with consumer attitudes specifically addressed in the cited literature, but are not treated as primary food categories in this review. “Fortified” or “functional” foods are referenced in the context of consumer health-motivation research but are distinguished from novel foods proper. “Cellular agriculture” refers specifically to the production of cultured meat and seafood. “Precision fermentation” is distinguished from traditional fermentation (e.g., yogurt and bread) in that it uses genetically modified host organisms to produce targeted ingredients. This distinction carries significant implications for consumer perceptions of naturalness and trust.

## 2. Toward an Integrated Framework of Novel Food Acceptance

In this review, the integrated conceptual framework was developed through an integrative synthesis of the existing literature on consumer acceptance of novel foods. Four interconnected and recurring themes consistently emerged across studies, such as cultured meat, plant-based meat products, algae-based foods, insect protein, and precision fermentation-derived foods. These themes, behavioral economics, sensory science, food policy, digital and AI-driven marketing, were recognized as the principal domains affecting consumer acceptance. This framework presents an evidence-based integration of the available literature, with insights explicated using established innovation adoption and consumer behavior theories to offer a detailed interdisciplinary perspective on the acceptance of novel foods. This section presents an integrative conceptual framework that synthesizes findings from all four domains, identifies points of convergence and contradiction, and articulates the moderating role of cultural context, product familiarity, and sociodemographic factors.

At the core of the framework is the premise that consumer acceptance of novel foods is neither purely sensory nor purely cognitive. Sensory evaluation research consistently demonstrates that hedonic responses to taste and texture are primary gatekeepers of novel food trial. If a product fails to meet minimum sensory expectations, no amount of information-based persuasion or environmental framing is sufficient to drive repeat purchase. However, sensory acceptability alone does not predict sustained adoption, as consumer acceptance of novel foods is also shaped by multiple interrelated factors, including perceived health benefits, food neophobia, trust in food technologies, perceived naturalness, cultural norms, environmental concern, price sensitivity, and social influence [12,13,14]. Behavioral economics research demonstrates that even sensorially acceptable novel foods face significant adoption barriers rooted in status quo bias, loss aversion, and food neophobia [15]. Consumers exhibit a persistent status quo bias, perceiving dietary changes as likely losses rather than prospective gains. In addition, they systematically value convenience and short-term palatability over delayed benefits, including health outcomes and environmental sustainability.

A critical interaction exists between the sensory and behavioral economics domains: familiarity through repeated exposure reduces both perceived sensory novelty and neophobic resistance simultaneously. This finding has direct implications for AI-driven personalization strategies: by tailoring product recommendations and exposure sequences to individual flavor profiles and prior food experiences, digital marketing tools can effectively accelerate the familiarity curve [16]. Evidence from social media listening studies suggests that exposure to positive peer reviews of novel food products on platforms such as TikTok and Instagram reduces perceived disgust and increases stated willingness to try, particularly in younger consumer segments. However, a significant contradiction exists in this literature: while digital exposure increases awareness and curiosity, this heightened willingness to try does not reliably translate into actual purchase behavior or repeated consumption, particularly for insect-based and cultivated meat products, where the “yuck factor” resurfaces in real-world purchase contexts even after positive virtual engagement [14]. This discrepancy underscores the critical need to distinguish between stated preferences expressed in online environments and concrete purchasing decisions, as the former may reflect aspirational or socially desirable responses rather than genuine behavioral commitment [1,12].

The policy dimension interacts with both behavioral economics and sensory science in important ways. Mandatory allergen labeling for insect proteins in the EU has been shown to both protect vulnerable consumers and inadvertently amplify risk perceptions among non-allergic consumers, reducing purchase intent independent of sensory evaluation [17]. Conversely, clean-label and minimal-processing claims that align with the “naturalness” heuristic can partially offset neophobic resistance in PBMA and precision fermentation products [12]. The policy–trust nexus is particularly salient for cultivated meat: regulatory approval communicates institutional endorsement, which in cultures with high trust in government and scientific institutions (e.g., Singapore, which granted the world’s first regulatory approval in 2020) significantly improves consumer confidence, whereas in low-trust contexts, the same regulatory framing may increase suspicion of industrial manipulation [18].

Cultural context operates as the most pervasive moderating variable across all four domains. The same product, sensory profile, marketing message, and policy frame can produce markedly different acceptance outcomes across cultural settings. Insect consumption, for example, is embedded in the culinary traditions of approximately two billion people globally, primarily in Asia, Africa, and Latin America, where acceptance rates are significantly higher than in Western Europe and North America, where insect eating carries strong disgust associations [19]. Similarly, responses to precision fermentation and genetic modification disclosures are moderated by cultural orientations toward naturalness and technological trust, with notable differences observed between Scandinavian, Southern European, and East Asian consumer populations [12]. This cross-cultural heterogeneity is a key source of contradictory findings in the literature. It must be accounted for when generalizing results from single-country studies, a limitation that affects the majority of studies included in this review, which skew toward Western, educated, urban, and relatively affluent samples.

In summary, the integrated framework proposed here suggests that effective novel food adoption strategies must operate simultaneously across sensory, psychological, digital, and policy levers. The reviewed literature does not present a consistent narrative but rather a context-dependent, method-sensitive body of evidence. This is because a consistent discrepancy arises between stated willingness to try and actual consumption behavior, with positive attitudes reported in surveys often not translating into real-world purchasing decisions. This inconsistency shows the limitations of self-report and the significance of behavioral and experimental confirmation. Moreover, drivers of health and sustainability play dual and even conflicting roles, as they can be both motivators and sources of skepticism depending on product framing and perceived naturalness. It must be calibrated to the cultural and sociodemographic context of the target population. The quality of the evidence supporting these findings varies across the methodological approaches. Experimental and sensory-based research studies offer more concrete indicators of actual behavior, though they are often conducted in controlled settings and may introduce some hypothetical bias. Survey-based research studies provide broad attitudinal information, but are subject to hypothetical bias. Cross-cultural research offers greater generalizability; however, it is relatively limited in number, which can introduce variability and sometimes lead to inconsistent findings. Optimizing a single dimension in isolation, such as improving taste without addressing food neophobia, or implementing AI-based personalization without adequate regulatory legitimacy, is unlikely to produce sustained, large-scale behavioral change.

## 3. Novel Foods: Drivers and Challenges in Consumer Preferences

Consumer preferences for novel foods are shaped by a range of complementary factors, including evolving attitudes toward health, sustainability, and innovation [20]. These preferences are influenced by psychological and cultural factors, as well as emerging value-based food movements such as slow food and organic food consumption patterns. Understanding these drivers is vital to predicting consumer acceptance and market trends in the rapidly evolving food landscape. Growing concerns about personal well-being, animal welfare, and climate change have driven demand for cultivated meat, insect-derived products, and plant-based products [13]. These trends are also boosted by increasing market demand for functional foods (lower in sugar, salt, or fat, higher in protein, or with functional additives), especially those with improved health or nutritional benefits [3]. Technological advancements, such as genetic modification, precision fermentation, organic biotechnology, and 3D printing, have presented new opportunities; however, consumer acceptance varies based on observed trust and perceived naturalness [12]. Digital food media and globalization have also increased exposure to culturally and ethnically diverse cuisines, contributing to food neophilia in some cases [21].

**Figure 3:**
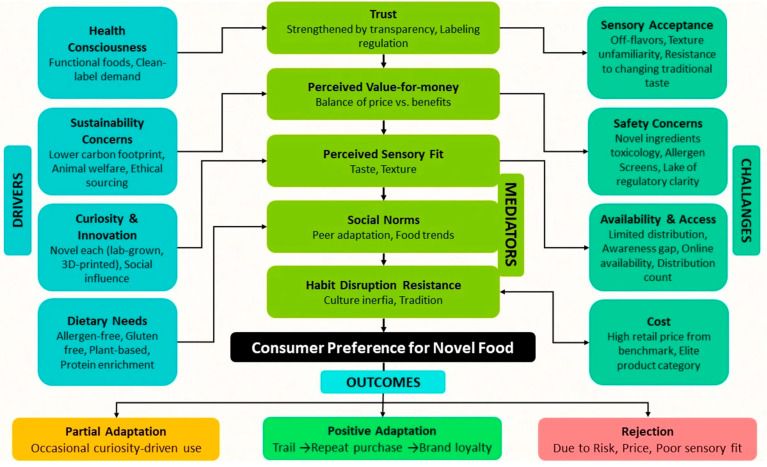
Consumer preference mechanistic model for novel foods to describe the key drivers, challenges, mediating factors, and adoption outcomes. The frame is a synthesis of the main determinants identified in a semi-systematic narrative review of the literature, as well as the major drivers (health, sustainability, curiosity and innovation, and dietary needs) and challenges (sensory attributes, safety concerns, and cost/access) that influence consumer preferences. The elements included were chosen based on consistency in reporting and applicability across previous empirical and review studies, with highly product-specific or regionally confined factors excluded to ensure overall applicability. The mediating constructs are trust (e.g., transparency and brand reputation), perceived safety (e.g., regulatory approval), and perceived value for money and social norms, which collectively influence consumer attitude formation. Such interactions eventually affect adoption outcomes, including positive adoption (repeat purchases and brand loyalty) or partial adoption. The figure provides an integrative overview of the most apparent themes that emerge throughout this review.

A critical review of this literature reveals important heterogeneity in findings that purely descriptive summaries tend to obscure. First, the magnitude of neophobia effects differs substantially across product categories: insect-based proteins consistently elicit the strongest disgust and rejection responses in Western samples, with acceptance rates in experimental tasting studies typically ranging from 18 to 43%, whereas plant-based meat alternatives achieve substantially higher acceptance (55–78%) in the same populations, largely because they more successfully mimic the familiar sensory profiles of conventional meat [14,22]. Second, methodological differences across studies substantially affect reported acceptance rates: survey-based studies that present novel foods abstractly yield significantly lower willingness-to-try scores than studies that employ actual-tasting protocols with anonymized products, highlighting the well-documented gap between stated and revealed preferences [23]. Third, health motivation operates as both a driver and a barrier depending on product framing. When plant-based meat alternatives are positioned as “healthy,” they attract health-motivated consumers but simultaneously trigger skepticism among consumers who associate highly processed products with poor nutritional profiles [1]. These contradictions highlight that consumer acceptance is not linear but rather highly context-dependent, influenced by the product type, framing strategy, and study methodology. Specifically, a discrepancy between abstract survey data and actual-tasting data has been observed, suggesting that multi-method approaches should be used to integrate behavioral, sensory, and experimental data to enhance reliability. Figure 3 represents a preliminary conceptual framework developed a priori to guide the scope and organization of this review. The elements included in the framework, health and nutrition, sensory attributes, sustainability, cultural and psychological factors, technological innovation, ethics, digital influences, and pricing, were selected based on recurring themes identified during the initial pilot literature search and are consistent with established domains in the consumer food choice literature [21]. No elements were systematically excluded; however, the framework does not claim exhaustive coverage of all possible determinants. Rather, it serves as a heuristic organizing structure to facilitate the narrative synthesis presented in subsequent sections.

### 3.1. Health and Nutrition

Health is a primary driver of consumer preferences and acceptance of novel foods. Still, current trends reveal a shift from awareness of essential nutrition to a fascination with functional, preventive, and personalized nutrition [24]. Only the adequacy of macronutrients no longer justifies modern consumers’ food preferences; they now strive for foods that confer distinct metabolic and physiological effects, including improved immune resilience, gut microbiota composition, cardiometabolic health, and cognitive function. Macronutrients include carbohydrates, the body’s primary energy substrate at 4 kcal/g, fueling brain and muscle function; proteins essential to tissue synthesis, enzyme production, and immune function at 4 kcal/g; and dietary fats critical for fat-soluble vitamin absorption, hormone synthesis, and concentrated energy storage at 9 kcal/g [25]. This trend has driven the development of functional foods enriched with antioxidants, bioactive compounds, dietary fiber, omega-3 fatty acids, prebiotics, and probiotics, which are practically correlated with systemic health regulation and disease prevention [26,27].

The similar rise of cultured meat, alternative fats, and plant-based proteins (e.g., pea and soy isolates) indicates a broader consumer shift toward health-optimizing protein sources with lower cholesterol content and reduced saturated fat [28]. Innovation in food biotechnology, which facilitates the production of nutrient-rich foods through cellular agriculture, synthetic biology, and precision fermentation, is a concurrent response to this demand. These innovations enable the design of clean-label vitamins and proteins with improved bioavailability while minimizing the use of unhealthy additives commonly found in processed foods, for example, vitamin C (ascorbic acid: antioxidant, collagen synthesis, and immune defense), vitamin D (calciferol: calcium absorption, bone mineralization, and immune modulation), and B vitamins, including B12 and folate (energy metabolism and neurological function, notably absent from most plant-based diets, making their bioavailability in novel foods a critical nutritional consideration) [29]. The term clean label refers to products formulated without artificial additives, preservatives, or synthetic ingredients, as defined by consumer-facing labeling standards rather than a regulatory category [30]. Similarly, unhealthy additives should be understood to refer to ingredients associated with adverse health outcomes in the epidemiological literature, such as synthetic emulsifiers, artificial colorants, and high-fructose corn syrup, acknowledging that unhealthy remains context- and dose-dependent [31]. Likewise, incorporating genome-informed dietary approaches through microbiome and nutrigenomics-targeted food methods adds a layer of specificity to contemporary nutrition, supporting the concept that “one-size-fits-all” nutritional policies are increasingly outdated. It is important to acknowledge, however, that such personalized approaches carry significant equity implications. Genomic testing, microbiome sequencing, and nutrigenomic profiling currently require access to advanced diagnostics and incur considerable costs, making them accessible primarily to individuals of higher socioeconomic status and thereby risking exacerbating existing health inequalities across social groups and world regions [32]. Furthermore, the clinical evidence bases for personalized nutrition recommendations derived from genomics and microbiome data remains limited and insufficient to justify wholesale abandonment of population-level dietary recommendations, which retain important advantages of low cost, scalability, and universal accessibility. The statement that one-size-fits-all nutritional policies are outdated should therefore be qualified: personalized nutrition is a promising complementary direction rather than a replacement for evidence-based public health nutrition guidance [33].

Although scientific advancements have boosted the prospect of novel foods providing quantifiable health benefits, consumer trust remains a vital moderating factor. Research reveals that perceived labeling transparency, the naturalness of food enrichment strategies, and ingredient familiarity affect consumer preference and acceptance [34]. Functional food components are more readily accepted via naturally healthy carriers, such as fiber-enriched cereals and yogurt, than via indulgent carriers, such as calcium-enriched juice, ice cream, or chocolate. Similarly, integrating nutrients in “natural “ products, such as vitamin D-fortified milk, is valued more positively than incorporating them into highly processed food products (e.g., sausage or fish), reflecting an intrinsic consumer preference for minimal manipulation and food authenticity [35].

Moreover, the increasing demand for clean-label foods, such as those free from artificial preservatives, genetically modified organisms (GMOs), and synthetic additives, shows how ethical and emotional references are often linked to health motivations [36]. Although engineered health solutions are assured, skepticism remains high, particularly when food inventions are perceived as unnatural or technical. It emphasizes the necessity of science communication approaches, underscoring efficacy, safety, and their connection to consumer values [37].

Today, health-driven food preferences define a dynamic interplay of socio-cultural ethics, consumer psychology, and scientific innovation. As preventive nutrition, personalization, and sustainability become central pillars of the modern food system, addressing consumer health perceptions will remain essential to successfully adopting novel food technologies.

### 3.2. Sensory Attributes

Novel foods, such as plant-based meat, insect-based products, lab-grown meat alternatives, algae-based foods, and precision-fermented products, often face sensory challenges in terms of consumer acceptance. These challenges are primarily associated with a product’s smell, taste, appearance, and texture [38]. Beyond objective sensory attributes, cognitive factors, particularly emotions and autobiographical memories, are equally potent determinants of consumer preference and rejection. Insect-based foods frequently evoke negative emotional associations, including disgust, fear, and anxiety rooted in cultural memories linking insects with disease, poverty, or contamination [39]. Research using emotion lexicons such as EsSense25 demonstrates that sensory attributes map onto emotional descriptors: sweetness may evoke “joy”, while bitterness maps to “disgust” [40]. Consumer strategies must therefore address emotional and memory-based barriers alongside sensory optimization. Sensory characteristics are essential because they significantly affect consumers’ willingness to try a novel food and their first impression of it. Even if products align with consumer preference values, such as animal welfare and sustainability, failure to meet sensory expectations can lead to rejection [21].

It is particularly evident in plant-based meat analogs (PBMAs), which aim to replicate the sensory attributes of animal meat. Historically, multiple products have failed to replicate the meat’s savory flavor, mouthfeel, and juiciness, ultimately creating a perception of imperfect texture and taste. In a systematic review, Appiani et al. [41] reported that intrinsic sensory attributes are key to developing consumer willingness to try, even overshadowing sustainability or health benefits. Likewise, PBMAs have significantly advanced flavor and appearance through heme-mimetic ingredients and extrusion techniques; however, reproducing the complex matrix of animal meat remains challenging [42].

Moreover, insect-based foods face sensory limitations, particularly in terms of texture and visual appeal. Lamers [43] reported that consumers rate insect-based proteins lower in sweetness, texture, and taste than insect-free proteins, revealing a pronounced negative preference caused by textural and visual cues. The sensorial “ick factor” usually overcomes rational acceptance, even among environmentally conscious consumers. Olsen et al. [44] reported that high levels of microalgae inclusion in food products can result in strong “fishy” flavors or a green color, leading to a preference for lower inclusion levels. Verardi et al. [45] demonstrated that consumers acceptance of insect-based food is significantly affected by information disclosure, with changes in visual attractiveness shaped by attitudes, knowledge levels, and gender-specific sensitivities.

Furthermore, cross-national perception studies reveal that sensory and emotional feedback, rather than objective knowledge, are the primary factors influencing consumer acceptance. A cross-national survey found that unfamiliar sensory characteristics of food triggered emotional discomfort and were a potent predictor of rejection rather than environmental concerns or health risks [37]. Children with food neophobia tend to decline to consume novel foods due to their unwillingness to experience new tastes and sensory experiences, as well as their perception of non-familiar foods [46].

Recent strategies to address these sensory concerns include advanced formulation approaches, flavor enhancers, natural colorants, structured plant proteins, and the introduction of novel ingredients in traditional formats such as baked goods and snacks [42]. Moreover, guided tasting sessions, sensory exposure, and consumer education are emerging tools to enhance sensory acceptance and gradually decrease food neophobia [21]. Ultimately, novel foods have gained preference for sustainability and technological advancement; their market viability depends on meeting sensory expectations. Producers can address sensory issues regarding novel foods by enhancing flavor profiles, improving texture, and masking undesirable off-notes, also applying novel processing techniques and incorporating iterative consumer sensory evaluation to align product properties with consumer expectations.

### 3.3. Sustainability and Environmental Concerns

Sustainability has evolved into a compelling and multidimensional driver, increasing consumer preference for novel foods as individuals pursue dietary alternatives that align with their environmental importance [15]. Nowadays, sustainability incentives have shifted from public concern to personal commitment, with consumers increasingly using their food choices to express ethical values and contribute to climate action. This shift is particularly evident among urban, younger, and environmentally literate populations. Furthermore, elevating eco-certifications, carbon labeling, and life-cycle assessment (LCA) transparency enhances the accuracy and transparency of consumer sustainability claims [47].

Climate change is increasingly shaping consumer preferences as individuals recognize the impact of food production on environmental issues. Increasing awareness of the ecological footprint of traditional animal agriculture, including water depletion, land use, and greenhouse gas (GHG) emissions, has led many consumers to adopt more eco-conscious diets, often substituting meat with lab-grown, insect-based, and plant-based options [16]. The global food system accounts for approximately one-third of greenhouse gas (GHG) emissions, with livestock farming significantly contributing [48]. Reducing the consumption of animal-source food and replacing it with plant-based or alternative proteins can help lower greenhouse gas (GHG) emissions. Dietary shifts can reduce food-related emissions by up to 80% without sacrificing nutrition [49,50]. For example, lab-grown meat reduces GHG emissions and improves resource efficiency compared with traditional livestock farming, which can appeal to environmentally and ethically conscious consumers [51].

It is important to contextualize these macro-level figures within consumer acceptance research. The GHG estimate cited above (approximately one-third of global anthropogenic emissions) is drawn from global life-cycle assessment (LCA) modeling by Crippa, Solazzo, Guizzardi, Monforti-Ferrario, Tubiello and Leip [48], covering all world regions and food system stages from production to retail. The 80% emission-reduction potential reported by Mazac, Meinilä, Korkalo, Järviö, Jalava and Tuomisto [50] refers specifically to modeled European dietary transitions toward novel food-inclusive diets and is not generalizable to all geographic and dietary contexts. These statistics are presented here not as standalone environmental facts but because growing consumer awareness of food’s environmental footprint has consistently been identified as a motivator for novel food trials, particularly among younger, urban, and environmentally literate populations [15,16]. The strength and generalizability of this link between macro-level environmental awareness and individual-level purchase behavior, however, remain heterogeneous across cultures and income groups, as discussed in subsequent sections.

The global population is expected to reach approximately 9.7 billion by 2050 under medium-variant scenarios. This demographic growth will increase global food demand by mid-century, ranging from 50 to 60% compared with current levels, thereby placing significant stress on global food production systems and ecosystems [52]. In response, plant-based and technology-driven foods are being promoted as providing environmental benefits by reducing global warming, land use, water use, and freshwater eutrophication by up to 88%, 83%, 87%, and 95%, respectively [53]. For example, insects such as crickets and mealworms are promising sources of protein. They require minimal resources, emit fewer greenhouse gases (GHGs), and offer a potential solution for food security compared with livestock [53].

Novel foods, such as precision fermentation-based proteins, microalgae, and cultured meat, are deemed sustainable due to their ethical production methods and lower resource intensity. However, consumer acceptance remains based on trust in technology and the authenticity of environmental claims [54]. Giacalone and Jaeger [37] conducted a multi-country survey to study consumer acceptance of novel food technologies in Australia, India, Singapore, and the United States. Although technologies such as cell-cultured meat and insect-based products offer sustainability benefits and advanced production advantages, they generally receive lower levels of consumer acceptance. In contrast, they found that minimally processed and natural foods, such as those from urban farming, were highly accepted. While environmental concerns are critical, familiarity and naturalness significantly influence consumer preferences and acceptance of novel foods.

Notably, recent studies reveal that sustainability concerns are more effective when aligned with price, health, and sensory attributes, suggesting that an integrated messaging approach is needed. Deconinck, Giner, Hobeika and Nauges [15] reported that understanding and trust in sustainability claims affect consumer behavior. An Organization for Economic Co-operation and Development study in 40 countries involving 37,000 consumers observed that while there is interest in sustainability declarations such as “organic,” “natural,” and “locally produced,” several elements impede their effectiveness. These products have unclear labeling, high prices, limited availability, and skepticism regarding their authenticity. The results highlight that clear communication and trust are essential for sustainability claims to influence consumer preferences effectively [15].

Achieving global sustainability requires transitioning to more sustainable food systems, such as edible insects, algae, and lab-grown meat, to mitigate the effects of climate change and ensure food security. Regardless, consumer acceptance is influenced by price, taste, and convenience. Explicit environmental affirmations are crucial to building trust, and food enterprises must balance sustainability, affordability, and quality to promote adoption.

### 3.4. Cultural and Psychological Factors

Cultural and psychological factors play a fundamental role in shaping consumer preferences for novel foods. These factors are deeply rooted in societal norms, traditions, and individual perceptions, which can vary significantly across regions and demographic groups [55]. Food choices are often intrinsically tied to cultural identity, social values, and personal beliefs, creating opportunities and barriers to the acceptance of novel foods. In many cultures, food is not just sustenance but also an expression of tradition, heritage, and belonging. As a result, unfamiliar or unconventional foods may face significant resistance if they deviate from established culinary norms [56].

Food neophobia, a psychological factor that causes hesitation in trying new or unfamiliar foods, is a significant barrier to consumption. Disgust and fear also contribute to rejection of novel foods, particularly when they deviate from traditional dietary norms. Monaco et al. [57] reported that emotions (disgust, fear, and anxiety) and socio-cultural factors (cultural norms, food taboos, and group conformity expectations) are influential barriers to consumer acceptance of cultured meat and insects. Curiosity and familiarity can lead to consumer acceptance, but overcoming initial preferences remains challenging. Novel foods based on familiar ingredients or flavors are less resistant to change than new products. For instance, plant-based burgers that mimic the taste and texture of beef are more readily accepted than unfamiliar alternatives. Familiarity helps reduce food neophobia and the perceived risks of trying new foods [58].

Cultural values and norms can influence food preferences, but their effects on novel food acceptance are complex and should not be generalized strictly along collectivist–individualist lines. Rather than uniform behavioral patterns, consumer responses are shaped by interacting factors, including food neophobia, trust, perceived risk, product familiarity, and social influence. For example, group influence and social norms may shape willingness to try novel foods, while individual differences in openness to experience and perceived benefits also contribute to acceptance across cultural contexts [59]. Therefore, cultural orientation should be considered one of several contextual factors affecting consumer acceptance of novel foods rather than a deterministic predictor. Onwezen, Bouwman, Reinders and Dagevos [14] reported, in a systematic review, that preferences for novel foods such as algae, cultured meat, and insects differ significantly across countries, influenced by cultural exposure and attitudes.

Lab-grown and plant-based meats are often met with skepticism in cultures where traditional meat holds cultural or culinary significance, as these choices may be perceived as a threat to identity. Likewise, edible insects are acceptable in Africa, Asia, and Latin America, where entomophagy is traditionally practiced, while in many Western territories, they are less accepted due to psychological discomfort, limited familiarity, and prevailing cultural norms. One important psychological factor affecting acceptance is food neophobia, which can reduce willingness to try insect-based products, although its effects may be moderated by factors such as prior exposure, product familiarity, and cultural background [58].

Consumers tend to oppose novel foods they perceive as unnatural or artificial, particularly those involving cellular agriculture or genetic modification. Acceptance increases when the benefits, such as sustainability and health, are comprehended and outweigh the risks, thereby establishing trust in technology [12]. Consumers readily accept functional additive-enriched olive oil or yogurt because they trust the label and its associated sustainable or ethical practices. Social context, reputation, and perceived authenticity play pivotal roles for local producers, whereas multinational brands often encounter skepticism associated with globalization and corporate concerns [60]. It emphasizes that understanding all factors is important for introducing novel foods successfully. Approaches, such as reducing food neophobia through education, culturally sensitive marketing, and aligning new products with cultural values, will likely improve acceptance rates.

### 3.5. Neophobia and Cultural Disgust

Food neophobia and cultural disgust, which are psychological concerns embedded in socio-cultural and evolutionary factors, notably affect acceptance of novel foods. Disgust, an instantaneous emotional response to dislike, typically arises from beliefs about the origin and nature of food and is shaped by cultural norms and values. For example, although insects are a conventional food in many cultures, others perceive them as disgusting, causing rejection of insect-based foods [61]. Neophobia is the unwillingness to eat novel foods; consumers with high neophobia tend to show solid disgust feelings and recognize higher risks linked with trying unfamiliar foods [62,63].

In cross-cultural studies, Thai consumers have shown greater acceptance of insect-based foods, as insect consumption is common in their culture. In contrast, Dutch consumers were less familiar with insect-based foods and showed higher levels of neophobia and disgust. This highlights that psychological factors, which vary across populations, play a significant role in shaping food acceptance through cultural familiarity and exposure [63]. Cross-cultural analyses further underscore the importance of tailoring approaches to distinct cultural contexts. A comparative study between Irish and Greek consumers revealed that Irish participants were more familiar with insect-based foods, likely because they were more readily available in local markets. These results underscore the necessity for culturally sensitive strategies when launching novel foods in distinct populations [63]. Ramírez-Rivera et al. [64] examined insect-based food neophobia among 620 Mexican consumers, finding that protein type and gender significantly influenced emotional responses, while protein type, consumer context, and gender significantly influenced memory recall. Cognitive mapping revealed that insect-based foods consistently elicited negative emotions (e.g., disgust and worry) and memories (e.g., poverty and disease) across genders, with men reporting stronger responses than women, and urban consumers recalling more memories that rural ones. These results show the need to address cognitive factors in acceptance strategies for insect-based foods. Escandón et al. [65] reported, in a cross-sectional study among Ecuadorian children and adolescents (n = 360), that willingness to consume insect-based foods was primarily driven by positive attitudes. In contrast, food disgust negatively influenced acceptance, with males showing a higher willingness to consume.

These concerns can be overcome by increasing familiarity through repeated favorable sensory experiences and exposure. For instance, integrating novel ingredients into regular foods can mitigate visibility and improve acceptance. Proper knowledge of the health and environmental advantages can influence consumer perspectives. Nevertheless, the usefulness of these approaches can vary depending on cultural contexts and individual differences [13]. Comprehending these psychological factors and implementing plans to address them by considering cultural contexts, providing informative cues, and increasing familiarity are necessary to encourage the adoption of sustainable and novel foods.

### 3.6. Technological Innovations and Perceived Naturalness

Technological innovations are crucial in food production, addressing challenges such as food security, sustainability, and resource efficiency. Advances in biotechnology, food engineering, and agriculture have led to the development of novel products, including lab-grown meat, algae-based proteins, and 3D-printed food, which aim to meet the growing global food demand while minimizing environmental impact [66]. Consumers’ understanding and trust in scientific advancements significantly influence their acceptance of these novel technologies [67]. Some consider these innovations compelling solutions to global challenges, while others remain skeptical due to concerns about safety, ethics, and the perceived unnaturalness of these solutions. Like lab-grown meat, precision fermentation products are viewed by some consumers as sustainable alternatives to conventional animal proteins, while others remain skeptical due to concerns about unnaturalness and safety [68]. This skepticism, typically rooted in fear and a misunderstanding, is a barrier to adoption. Advanced biotechnological processes, such as synthetic biology and fermentation, may face resistance due to safety and potential health concerns. However, consumer skepticism towards novel food technologies is not solely attributable to fear and misunderstanding. A substantial body of literature demonstrates that it can also reflect both emotional responses (e.g., disgust and perceived unnaturalness) and rational risk assessments grounded in concerns about food safety, long-term health effects, environmental consequences, and regulatory adequacy. Characterizing skepticism purely as a knowledge deficit risks misidentifying the intervention target and may lead to ineffective communication strategies [69]. Studies challenge traditional food concepts and raise ethical concerns, particularly regarding genetic manipulation and unconventional production methods [70].

Perceived naturalness is essential to shaping consumer choice to adopt novel foods. Consumers associate naturalness with sustainability, health, and safety, so they prefer foods that are more “natural” or less processed. This factor influences their preference for novel technologies, such as insect-based products, cultured meat, and genetically modified organisms (GMOs). For example, Wang et al. [71] recently reported that Chinese consumers are highly accepting of grass-fed dairy products, associating them with naturalness and revealing a willingness to pay a premium for these characteristics. Many cultures prioritize natural, minimally processed foods, believing them to be healthier and more authentic. Conversely, products perceived as highly processed or artificial may face resistance even if they offer sustainability and nutritional benefits [72]. Food producers and processors use novel nonthermal technologies to meet demand for quick meals, enhance shelf life, and maintain safety and flavor [73]. However, some consumers remain skeptical about processed foods, believing that they are harmful or unsustainable. Therefore, promoting technological benefits while reassuring users about safety and naturalness is necessary to overcome this perception [74]. Socioeconomic factors influence novel food preferences; these differences are more nuanced than a simple high- versus low-income distinction. Consumer responses vary across education, income, occupation, and food access dimensions. High socioeconomic groups prioritize sustainability, ethics, and health attributes, while lower socioeconomics groups prioritize nutrition, food safety, affordability, and accessibility. These variations can affect the pace and pattern of novel food adaptation depending on resource availability and perceived values [56].

Effective communication and education regarding novel food technologies, their benefits, and safety are essential to improving consumer acceptance [67]. Consumer education is vital as the demand for convenience and functional foods grows, accelerated by the COVID-19 pandemic. Studies conducted during COVID-19 lockdowns found that 15–42% of Europeans modified their food consumption patterns, shifting toward shelf-stable foods, increased home cooking, and health-promoting choices [75,76]. The pandemic also heightened awareness of food system fragility and accelerated interest in alternative proteins among previously disengaged consumer segments. Additional accelerants of dietary change include urbanization and the erosion of traditional food cultures, the proliferation of plant-based fast-food chains, and social media-driven food trends that normalize novel food consumption among younger cohorts [77]. Trusted third-party endorsements and marketing emphasizing environmental benefits, sustainability, and health advantages can boost confidence [51].

### 3.7. Ethics and Animal Welfare

Ethical considerations, particularly animal welfare-related ones, are becoming a primary driver of consumer preferences for novel foods, especially in ethically conscious and high-income societies. Conventional livestock is highly considered to conflict with contemporary ethical norms due to extreme farming conditions, animal moral status, and suffering [78]. The reviewed evidence suggests that consumers are willing to pay a premium for foods that meet higher animal welfare criteria. For example, Nakavachara et al. [79] analyzed real-world market (Swiss supermarket) data. They observed a 1-point increase in animal welfare outcomes (scale of 1–5), corresponding to a 16.5% price premium, with the most noticeable impact on egg and dairy products.

The recent literature has introduced the concept of “moral licensing,” in which consumers deem less sustainable behaviors or indulgences acceptable after making an ethical food choice [80]. This highlights the complex psychological dynamics influenced by ethical food preferences. Hyland et al. [81] reported three consumer groups: “The Indifferent,” “The Struggling,” and “The Engaged.” The indifferent reveal a low level of concern and occasional purchases. The struggling show concerns but faces availability and cost barriers, and the engaged vigorously pursue animal welfare-friendly foods. Ethical drivers also gradually bound intergenerational responsibility, food justice, and fair labor rules. Younger generations, especially Gen Z, often perceive their food preferences as acts of ethical and political expression. This transformation profoundly affects the novel food market, which is perceived as more humane and “cleaner”. Intergenerational transmission of food attitude represents another important mechanism shaping novel food acceptance. Parents serve as primary agents of food socialization, and children raised in households with positive attitudes toward plant diversity and sustainability-focused purchasing demonstrate persistently higher openness to novel foods in adulthood [82,83]. Food habits formed in childhood, even those rooted in cultural or religious identity, exhibit remarkable longitudinal stability, making early household-level and educational intervention a high-leverage point for novel food adoption strategies. Evidence for this claim varies by country and socioeconomic group: surveys in Western Europe and North America consistently find that Gen Z expresses stronger ethical and environmental motivations in food choices compared with older cohorts [1], but these patterns are less consistent in lower-income or non-Western contexts, where affordability and cultural tradition remain dominant decision drivers [14]. The association between novel food markets and production also deserves scrutiny, as the environmental and welfare footprints of precision fermentation, cell-cultured meat, and insect farming entail distinct trade-offs that do not uniformly favor novelty [84].

Ethical concerns remain key in novel foods, such as insect protein and cultured and plant-based meat alternatives, which offer potential “ethical relief” by reducing or eliminating animal harm and preserving meat-eating traditions by mitigating ethical barriers. Ammann et al. [85] found that consumers prioritize animal welfare over environmental concerns when selecting dairy and meat products, indicating that ethical concerns about animal welfare are a primary driver of food preference. Animal welfare and ethical concerns significantly influence consumer choices; personal values, availability, and price also shape purchasing decisions. Therefore, novel food producers should emphasize ethical considerations and ensure accessibility to enhance market success and consumer acceptance. It is important to note, however, that personal and hedonistic benefits (taste, convenience, and value for money) consistently emerge as the primary drivers of food purchase decisions, with animal welfare functioning as a secondary motivator that becomes salient mainly in specific consumer segments. Ethics-centered marketing achieves greater success when paired with sensory and personal benefit messaging.

### 3.8. Digital and Social Influences

In the digital age, online platforms featuring influencer content, food bloggers, and social media have a powerful influence on consumer preferences for novel foods. These platforms enable individuals to quickly share trends, opinions, and experiences, leading to both positive and negative statements about novel foods. The effectiveness of influencer endorsements, however, is subject to important boundary conditions regarding source credibility, audience trust, and disclosure of commercial relationships [86]. Endorsements perceived as paid promotion elicit reactance and reduced persuasion, particularly among high-skepticism audiences. Heterogeneous audience segments respond very differently based on food involvement and prior product familiarity, necessitating segment-specific influencer strategies rather than assuming that influencer content uniformly reduces perceived risk and increases willingness to try new foods [87].

A new concept of digital influence is “digital food citizenship,” a way to express individuality through online food choices. A concept referring to participation of active, values-driven consumers in food-related discourse and decision making through digital platforms, including sharing sustainability content, participating in food activism, influencing peer food choices via social media, and engaging with food brands through online communities. Consumers make buying decisions based on product characteristics and how those choices are known within their social media circles. For instance, consumers choose lab-grown or plant-based meat not for its health benefits but to demonstrate their support for ethical or environmental causes. These preferences, which are often shared online, can influence others and create social pressure to make similar decisions [88].

Online platforms, such as social media, often reflect and reinforce social norms, significantly influencing food preferences. Social media influencers (such as those on YouTube, Facebook, TikTok, and Instagram) play a crucial role in introducing and normalizing novel foods. Their endorsements can reduce the perceived risks associated with novel and unfamiliar foods, thereby improving the willingness to try them [89]. These visual platforms also highlight the aesthetic appeal of food, which can influence preferences and perceptions. High-quality videos and images of novel foods can improve mental flexibility, making them appear more acceptable and familiar. Visual orientation reduces the cognitive effort required to process new information, thereby fostering a positive attitude towards novel foods [90]. Additionally, user-generated content, such as personal experiences and reviews, is shared online as social evidence and can help validate consumer assertions [87]. The literature indicates that trusted endorsements from digital influencers and personalities can significantly decrease skepticism for novel and unfamiliar foods, such as fermentation-derived dairy or insect-based protein [91].

Another advanced trend is the use of AI-driven personalization and algorithmic recommendation systems to shape food choices. AI-driven personalization offers significant potential to improve acceptance of novel foods; however, key limitations include data privacy concerns, algorithmic bias, and issues of data quality and representativeness. Additionally, limited transparency of AI models may reduce consumer trust, while a persistent gap often exists between digital engagement and actual food consumption behavior [92]. Based on past behavior, even smart fridges, e-commerce platforms, and food delivery apps encourage consumers to choose more health-oriented, sustainable, and novel foods [93]. Personalized, gentle hints can effectively change consumption practices without conscious thought by leveraging behavioral science in digital formats. Regardless, digital platforms also pose risks of misinformation and negative presentation. For instance, fermentation-derived products are associated with “synthetic” and lab-grown meat, which carries risks of “unnaturalness” and can quickly gain and undermine acceptance [94]. It is also noteworthy that sensory and consumer research has increasingly migrated to digital platforms. Online sensory panels conducted via video call platforms (e.g., Zoom-facilitated home tasting kits) have enabled geographically diverse data collection [95]. Social media platforms have been used to deploy Check-All-That-Apply (CATA) questionnaires and flash profiling tasks via Instagram Stories polls, enabling large-scale elicitation of consumer emotional and sensory responses to novel food images. TikTok food challenges and YouTube taste-test videos constitute unstructured sensory data that AI-powered sentiment analysis tools can mine to identify dominant flavor associations, disgust triggers, and novelty appeal across consumer segments [96]. Thus, digital content regulation and literacy are essential to intervention.

### 3.9. Pricing and Accessibility

The price of novel foods significantly determines consumers’ adoption or willingness to try them, particularly when perceived as risky or unfamiliar [97]. The market penetration of novel foods continues to grow, as consumers increasingly believe that they offer health benefits, with a growing emphasis on lifestyle wellness. Various products quickly fail in the marketplace due to pricing issues, which remain a significant obstacle to adoption [98]. Consumers usually weigh the price of novel food against its known advantages, but if it is substantially more costly than traditional choices, they may reject it regardless of its benefits. Rovai et al. [99] found, in a consumer-based study in the United States, that potential early adopters would purchase insect-based foods if the costs were equivalent to those of traditional alternatives.

Consumers often associate a higher price with technological advancements and improved quality. This perception is mediated by consumer familiarity and the degree of novelty. A survey by Herbert and Beacom [100] found that consumers perceive high-priced mealworm-based burgers as of higher quality than low-priced burgers. It raised concerns that low prices could undermine perceptions of quality. Narayana et al. [101] found that Sri Lankan consumers primarily base their purchase decisions on cost considerations. Consumer readiness to pay higher prices for new functional foods remains constrained, as research indicates that Chinese consumers are unlikely to accept expenses exceeding 40% above standard rates [102]. Demographics and economic status significantly affect price sensitivity. High-income consumers are willing to pay a premium for novel foods associated with animal welfare and environmental consciousness. On the contrary, lower-income buyers are price-sensitive and require stronger motivation to switch. Marketing practices that address price concerns have also grown. For example, initial pricing and associating novel foods with familiar products have been shown to increase trial rates [103].

The availability of novel products is also vital to shaping consumer preferences. Availability is not merely physical existence in retail environments but rather the ease of visibility, access, and consistency at the point of purchase. Researchers have acknowledged the availability of novel foods and have called for integrating and normalizing them into habitual consumption routines [104]. Szenderák et al. [105] conducted a systematic review. They found that despite health and environmental motivations, the adoption of plant-based meat is restricted by taste preferences, higher prices, and limited availability.

Behavioral aspects are also important in consumer acceptance. Lack of familiarity and food neophobia can restrain acceptance. However, increased exposure and availability can help alleviate these concerns by reducing associated risks and enhancing familiarity [13]. Psychological factors, such as familiarity with novel foods, can affect acceptance. For instance, if these are available in a known or public place, such as a grocery store, the individual will feel more comfortable and willing to try them [21]. Retailers and manufacturers can utilize availability by strategically placing novel foods in prominent locations and highlighting them prominently to encourage purchase [106].

### 3.10. Communication and Marketing Strategies

Marketing strategies and communication significantly influence consumer acceptance of novel foods, shaping their behaviors, attitudes, beliefs, and perceptions. Effective communication can reduce neophobia and promote acceptance by addressing consumers’ behavioral barriers and cognitive biases [16]. Mosikyan et al. [107] recently emphasized that trust building, transparency, and tailored messaging affect consumer preferences regarding novel foods.

Marketing strategies that highlight the health and environmental benefits of novel food have enhanced consumer acceptance. For instance, highlighting the sustainability benefits of plant-based choices and insect-based foods can attract environmentally aware consumers. Addressing potential heuristics and cognitive biases contributing to resistance is essential [16]. A recent study in young Indonesian consumers found that subjective norms, positive attitudes, and environmental beliefs significantly influenced the purchase intent of cultured meat. It indicates that marketing brands focus on targeted messaging that aligns with social influences and consumers’ values, alongside sustainable practices, to ensure long-term survival [108].

Sensory branding engages multiple senses and can emotionally connect with consumers, making novel foods more appealing. Organizations like Starbucks utilize sensory branding to enhance customer experiences and foster brand loyalty. Media delineation is also important in shaping consumer acceptance of novel foods. Interpersonal communication, such as influencer endorsements and word of mouth, is more effective in reducing risk perceptions. Personal interactions enable direct feedback and clarification, thereby building credibility and trust. Matwick [109] media discourse analysis found that positive media (Singaporean) could affect social standards and improve acceptance of plant-based meats and insect-based protein.

It is important to tailor communication approaches to distinct audiences, as gender, age, education level, and income influence consumer behavior and attitudes toward novel foods. For instance, younger consumers are more inclined to try new food flavors and trends, suggesting that marketing measures be tailored to attract diverse demographic groups [110]. Public resistance to GM foods cannot be attributed solely to improper marketing or misinformation. Rather, it reflects a convergence of factors, including value-based judgments about naturalness and ethics, concerns over corporate consolidation and environmental risks, and varying levels of trust in scientific institutions and regulatory bodies. Media coverage has at times amplified these concerns, but the underlying skepticism is often rooted in legitimate personal, cultural, and socioeconomic considerations. Likewise, cultured meat has faced consumer resistance due to misconceptions and unfamiliarity with its safety and production process [111]. Novel food development relies on targeted communication strategies that highlight product benefits and address consumer concerns during adoption. Older consumers are more interested in new health-oriented food products because they actively focus on disease prevention and health maintenance when making product choices. Market success in novel food development demands strategic benefits communication to specific consumer groups [112].

Culturally sensitive marketing, communication, and transparency can effectively highlight the advantages of novel foods, thereby significantly increasing consumer acceptance. Marketers can promote the substantial and accurate benefits of novel food products across markets by addressing behavioral barriers and employing positive messaging [13].

### 3.11. Safety, Trust, and Naturalness

Concerns about the safety, trustworthiness, and naturalness of novel foods significantly affect consumer acceptance and often serve as psychological barriers to adoption. Trust is crucial to shaping consumer perspectives; a lack of transparency from regulatory bodies and producers can foster skepticism toward novel foods [71]. For example, the introduction of GMOs in European regions encountered significant resistance due to consumers’ skepticism about products that encourage these technologies. Likewise, people are cautious about adopting nanotechnology in food, usually suspecting the credibility and intentions of the industries behind it [113].

Safety is another critical factor; novel foods are associated with potential health hazards, mainly unknown long-term impacts. Herbert and Beacom [100] reported that insect-based food consumption in Western countries is limited due to concerns about microbial contamination and allergen concerns. Despite regulatory inspections that ensure safety, the recognized threat remains a substantial barrier.

Naturalness is inherently associated with consumer perceptions of food healthiness and quality. Multiple consumers correlate natural foods with being more environmentally friendly and healthier. Therefore, unnatural foods such as lab-grown meat are typically rejected by consumers [111]. Niu et al. [114] stated that the unnatural image of cultured meat negatively affects consumer acceptance. Instead of specifying the product’s nutritional value and safety, the absence of naturalness can discourage consumers. Overcoming these factors requires building trust by engaging stakeholders in the production process, maintaining transparent communication, and providing safety assessments. This can reduce skepticism. The naturalness of novel foods should focus on their similarity to traditional products and their environmental benefits. These multifaceted barriers to the acceptance of novel foods, including high prices, sensory limitations, psychological and cultural resistance, and safety concerns, pose significant obstacles to the mainstream adoption of these foods.

## 4. Insights and Interventions for Shaping Preferences

### 4.1. Consumer Behavior Research Tools

Consumer acceptance of novel foods is analyzed through various behavioral research methodologies, including implicit methods, explicit methods, comparative sensory methods, instrumental sensory devices, and advanced technologies such as immersive virtual environments, artificial intelligence (AI), and big data analytics. Each methodology offers a distinctive understanding of consumer perceptions and preferences, enhancing the marketing and development of novel foods, as shown in Table 1. The studies and methodological approaches listed in Table 1 were selected to represent the breadth of established and emerging methods used in consumer research on novel foods. Inclusion was guided by: (a) citation relevance and frequency within the 2020–2025 literature; (b) methodological diversity, ensuring representation across explicit (self-report), implicit (neurophysiological and behavioral), instrumental, comparative, and advanced technological approaches; and (c) direct applicability to novel food consumer research. The inclusion of choice experiments, discussed separately, complements this overview.

#### 4.1.1. Explicit and Implicit Methods

Explicit or self-reported methods utilize direct consumer feedback to assess intentions, beliefs, and conscious attitudes regarding novel foods through structured interviews, questionnaires, and surveys [115,116]. A multi-country survey was conducted to examine consumer acceptance of novel food technologies, using questionnaires to measure consumers’ willingness to try, situational appropriateness, and emotional associations across populations. Such tools are instrumental in determining the cultural and demographic factors that affect acceptance [37].

Implicit or unconscious methods are categorized into physiological and behavioral tracking approaches and neurophysiological methods [117]. Neurophysiological methods estimate nervous system responses and brain activity (e.g., facial electromyography, functional magnetic resonance imaging, and electroencephalography) to provide insights into emotional (e.g., arousal and liking) and cognitive (e.g., memory and attention) connections. Physiological and behavioral tracking measures observable behavior (e.g., approach–avoidance actions and gaze patterns) and physiological signals (eye movements, heart rate, and skin conductance) to demonstrate preferences and unconscious reactions through biometric measurements, virtual reality (VR), and eye-tracking tools [118]. VR conditions have been used to stimulate shopping experiences, enabling researchers to monitor consumer behavior while controlling for variables and realistic elements. Eye-tracking analyses have demonstrated that visual awareness of nutritional information on packaging can significantly affect purchase intent and perceived healthiness [119]. Biometric measurements, which include facial expression analysis, galvanic skin response, and heart rate variability, reveal emotional engagement, stress, and arousal. These tools reveal automatic reactions that self-reporting may not capture [57].

**Table 1:** The detailed characteristics of consumer behavior research tools for novel foods.

Tests	Type	Subtypes	Purpose	Data Type/Output	References
Explicit (Self-Reported) Methods	Surveys	Online	Collect consumer opinions, preferences, and attitudes	Quantitative (scores, ratings, rankings, and Likert scales)	[120]
		Offline			
	Focus Groups and Interviews	FGD	Gain deep insights into consumer perceptions through discussions	Qualitative (text, transcripts, and themes)	[121]
		Ethnography			
		Grounded Theory			
	Affective Tests	Hedonic	Measure the overall liking or pleasantness of a product	9-point hedonic scale (Likert-type ratings)	[122,123]
		Preference	Determine which product is preferred over others	Preference ranking and paired comparisons	[123]
		Projective Mapping	Understand consumer associations between products and attributes	Spatial data (maps and clusters)	[122]
		Napping	Identify sensory similarities/differences through product placement	Spatial data (distances and clustering)	[123]
	Traditional Sensory Analysis	Discrimination	Determine if products have perceivable differences	Pass/fail results, d-prime values	[122,123]
		Descriptive	Characterize sensory attributes and intensities	Sensory profiles (spider plots and intensity ratings)	[123]
	New Sensory Methods	CATA	Capture qualitative perceptions with a structured approach	Binary data (checked/not checked responses)	[122,123]
		Flash Profiling	Rapidly gather descriptive attributes from panelists	Word clouds and frequency counts	[122,123]
		RATA	Similar to CATA but with intensity ratings	Ordinal data (intensity scales)	[123]
Implicit (Unconscious) Sensory Methods	Neurophysiological Techniques	EEG	Measure brain activity in response to stimuli	Brain signals (waveforms and neural activation maps)	[124]
		MEG			[124]
		SST			[117]
		fMRI			[125]
		fNIRS			[126]
		PET			[117]
		ECG			[127]
		TMS			[117]
	Physiological and Behavioral Tracking	Eye Tracking	Understand attention and focus on product attributes	Heatmaps and fixation duration	[128]
		fEMG	Measure facial muscle activity linked to emotions	Muscle response signals	[127]
		Electrodermal Activity	Measure emotional arousal through skin conductivity	Conductance levels (microSiemens)	[127]
Instrumental Sensory Devices	e-Nose	-	Analyze volatile compounds for aroma profiling	Chemical sensor signals	[129]
	e-Tongue	-	Detect taste differences in food products	Electrical response patterns	
Comparative Sensory Methods	PSP	Rating-Based PSP	Categorize consumers based on sensory preferences	Clustered preference data	[122,123]
		Triadic PSP	Compare three samples at a time for preference ranking	Pairwise preference rankings	
	PAE	DCEs	Evaluate consumer preference decisions under real-world constraints	Choice probabilities and utility scores	[122]
		BWS	Identify the most and least preferred attributes	Ranking data	
Dynamic Sensory Methods	Time–Intensity	DATI	Measure perception changes over time	Time-series intensity curves	[122,123]
		MATI			
	TCATA	-	Capture evolving sensory attributes over time	Binary time-series data	
Immersive Virtual Environments	Behavioral Simulation	VR Tasting Rooms and VR Shopping	Simulate real-world food purchasing to observe choice behavior	Engagement levels, gaze tracking, and behavioral logs	[10,119,130]
	VR Product Packaging Testing	A/B Testing in Virtual Shelves	Trust cues and test packaging appeal for novel food products	Selection logs and preference rankings	
	Facial Expression Recognition in VR	Emotion AI in VR Context	Determine emotional reactions to food, virtual tastings, or visuals	Emotion labels (disgust, surprise, and happiness)	
	VR-Based Eye Tracking	Heatmaps and Gaze Mapping	Determine attention to food labels, shelf placement, and packaging	Scan paths, fixation duration, and heatmaps	
Artificial Intelligence (AI) Based	AI Sentiment Analysis	Reviews, Social Media, and NLP	Access consumer attitudes regarding novel food (lab-grown and plant-based)	Emotion categories and sentiment scores	[131,132,133]
	Consumer Segmentation via Machine Learning	Unsupervised/Supervised Machine Learning	Cluster consumers based on preferences/behavior regarding novel food	Predictive scores and segments	
	AI-Powered Chatbots	Conversational User Experience	Elicit barriers and preferences toward new food via dialogue	Keyword clusters and conversation logs	
	Image Recognition of Meal Posts	CNN and Deep Learning	Determine types of food consumers prefer or share from photos	Metadata, food types, and labels	
Big Data Analytics	Big Data from Wearables and Smart Fridges	Sensor Fusion and IoT	Observe contextual and fundamental consumption factors influencing choices	Usage patterns and time-series data	[133]
	Geolocation Tracking	Proximity/GPS Sensors	Investigate visits to food events/restaurants related to novel food	Frequency patterns and spatial trails	
	Clickstream and Online Behavior Analysis	Web Usage Mining	Track user behavior on e-commerce platforms or food delivery apps	Clicks, product views, and navigation paths	

**EGD:** focus group discussion; **RATA:** Rate All That Apply; **SATA:** Select All That Apply; **SST:** Steady-State Topography; **fMRI:** functional magnetic resonance imaging; **fNIRS:** Functional Near-Infrared Spectroscopy; **fEMG:** facial electromyography; **PET**: Positron Emission Tomography; **ECG:** electrocardiography; **EEG:** electroencephalography; **MEG:** magnetoencephalography; **TMS:** Transcranial Magnetic Stimulation; **PAE:** Paired Attribute Evaluation; **PSP:** Paired Sample Preference; **DCEs:** discrete choice experiments; **BWS:** Best–Worst Scaling; **TCATA:** Temporal Check All That Apply; **MATI**: Multiple Attribute Temporal Intensity; **DATI:** Dynamic Attribute Temporal Intensity; **VR:** virtual reality; **NLP:** Natural Language Processing; **CNNs:** Convolutional Neural Networks; **IoT: Internet of Things; GPS: Global Positioning System**. Explicit methods (surveys/hedonic scales) have been applied to insect protein bars for taste acceptability testing and plant-based yogurt for texture evaluation. Implicit/neurophysiological methods (EEG, fMRI, and eye tracking) have been applied to lab-grown meat and algae-based snacks to assess automatic emotional and attentional responses during product exposure. Instrumental sensory devices (e-noses and e-tongues) have been applied to fermented plant proteins and cultured seafood analogues for aroma and taste profiling. Comparative sensory methods (PSP and DCE) have been applied to cricket flour bread and 3D-printed novel food formats to identify preferred sensory configurations. Dynamic sensory methods (TCATA and time–intensity) have been applied to precision-fermented dairy alternatives to track evolving taste perception during consumption. Immersive VR methods have been applied to insect-based snacks and cultivated meat products in simulated retail environments to observe purchase intention under realistic shopping conditions. AI-based sentiment analysis have been applied to social media discourse on plant-based meat and lab-grown meat launches to capture unprompted consumer attitudes. Big data/wearable analytics have been applied to track real-world purchase and consumption patterns of novel protein products via smart fridge and loyalty-card data.

#### 4.1.2. Instrumental and Comparative Sensory Devices

Instrumental sensory devices, such as electronic noses, e-tongues, and texture analyzers, measure the objective characteristics of novel foods, including flavor, aroma, and texture profiles [129]. These employ various analytical and measuring tools that produce consistent sensory data, surpassing the limitations of human sensory panels and human variability [134]. Installing machine learning algorithms in these devices enhances their analytical precision in sensory analysis, ensuring quality and consistency in novel food products [135].

Comparative methods are used to assess novel foods against familiar counterparts to estimate comparative consumer acceptance. Techniques such as descriptive analysis, hedonic scaling, and triangle tests are used to determine differences in overall appeal, texture, and taste [136]. Jaeger, Jin and Roigard [23] conducted a study on plant-based meat alternatives to compare sensory characteristics and consumer acceptance using comparative methods, aiming to identify the critical aspects that affect consumer choices. These comparative studies help refine product development and inform the creation of more effective design and marketing initiatives, ultimately better meeting consumer expectations.

#### 4.1.3. Advanced Technologies

Modern technologies have revolutionized consumer research behavior, including immersive virtual environments, artificial intelligence (AI), and big data analytics [133]. AI plays a crucial role in enhancing production methods, discovering novel proteins, refining product formulations for texture and taste, predicting consumer preferences, and developing innovative foods that replicate the flavor, texture, and nutritional profile of animal-based food. For example, AI-driven guidance systems can recommend personalized meal plans incorporating novel foods to enhance consumer acceptance and engagement [131].

Big data analytics enables the company to understand better consumer purchase motivations, decision-making processes, and behavioral patterns, thereby anticipating changes and trends in consumer preferences and behavior [133]. Immersive virtual environments, including augmented reality (AR) and virtual reality (VR), offer advanced platforms for evaluating consumer responses to novel foods in realistic settings, thereby enhancing the precision of consumer behavior analyses [130]. Personalization algorithms have significantly influenced consumer purchasing preferences by shaping individual dietary choices and health goals. These algorithms can suggest functional foods based on consumer demands, thereby improving the chances of adaptation. Likewise, transparency in an AI-recommended system fosters trust, which is crucial to consumer acceptance of novel foods [132]. Integrating AI in sensory studies has enhanced the efficiency and objectivity in assessing consumer feedback on novel food products. These technologies offer a comprehensive understanding of consumer acceptance and product development, which is crucial to achieving market success. Continuous evolution will play a vital role in shaping the future of novel foods and consumer preferences.

While big data and AI offer significant potential for advancing consumer understanding and personalized food recommendations, several limitations should be acknowledged. These techniques, based on robust model validation, may be limited in generalizability and prone to overfitting if not externally validated. Moreover, the use of biometric and behavioral data raises significant consumer privacy and ethical concerns, particularly regarding data storage, misuse, and consent. Data representativeness is another key challenge, particularly for sensor and digital-based datasets. Finally, practical implementation in food consumer research remains constrained by cost, technical expertise, and integration barriers across multidisciplinary systems [137,138].

#### 4.1.4. Choice Experiments and Willingness-to-Pay Studies

Choice experiments (CEs) and conjoint analysis are widely used methodological approaches in consumer research on novel foods, which merit a dedicated discussion. Unlike surveys, which elicit stated preferences, CEs present respondents with a series of hypothetical product profiles that differ across attributes (e.g., price, production method, label, and origin) and require them to choose their preferred option. This stated-preference design allows researchers to decompose the relative importance of individual attributes and estimate willingness to pay (WTP) for specific product characteristics. A critical methodological limitation of stated-preference WTP studies is hypothetical bias: consumers systematically overstate their willingness to pay in hypothetical choice tasks compared with actual purchase behavior. This bias is particularly pronounced for unfamiliar novel foods. Furthermore, choice experiments, conjoint analysis, and hedonic pricing capture distinct dimensions of value and are not directly comparable. A more integrated methodological framework that explicitly maps relationships between these approaches would strengthen the contribution of this research area [23].

Several recent studies have employed CEs to examine consumer preferences for novel foods. Caputo et al. [139] used a choice experiment integrated with sensory evaluation to demonstrate that flavor and texture similarity to conventional meat were the most valued attributes of plant-based meat alternatives, outweighing sustainability and price benefits. Zollman Thomas et al. [140] applied discrete choice modeling across Singapore, Germany, and the USA to show that price sensitivity and health claims differentially influenced WTP for precision fermentation-derived egg proteins across cultures. Nakavachara, Thongtai, Chalidabhongse and Pharino [79] analyzed Swiss market data using hedonic pricing to quantify consumer WTP premiums associated with animal welfare certification, finding a 165% premium per welfare scale point for egg and dairy products. These studies collectively highlight that consumer preferences are multi-attribute and context-dependent and that WTP estimates vary substantially with framing, income, and cultural background, findings that are directly relevant to pricing and communication strategies for novel food producers.

### 4.2. Behavioral Economics and Nudges

Nudging and behavioral economics approaches notably influence consumer preferences and perceptions of novel foods, such as insect-based products and cultured meat, by addressing emotional responses and intrinsic cognitive biases [57]. Consumers typically exhibit a status quo bias, diverging from their typical dietary routines, which they perceive as a likely loss rather than a gain. In which convenience and immediate taste are preferred over long-term advantages such as health benefits and environmental sustainability [13].

Nudging, an idea rooted in behavioral economics, provides subtle guidance that influences consumer preferences without restricting consumer choice. For instance, altering default options can lead to significant changes in behavior. In a field study, Onwezen, Bouwman, Reinders and Dagevos [14] reported that a Dutch restaurant redesigned its menu to make plant-based meat alternatives the default option, thereby significantly increasing consumer preference for them. Orders for seaweed-based alternatives increased by 16.5–58.3%, and orders for bean-based alternatives increased by 8.6–80.0%. This phenomenon highlights the influence of default settings on consumer perception and preferences for novel foods. Framing impacts are also essential and reveal novel food information about possible losses, such as environmental harm, by not trying sustainable novel foods; instead, gains can be convincing due to consumers’ loss-aversion preferences. Additionally, exploiting social norms by showing that several people approve of or adopt novel foods can enhance overall consumer acceptance, as many individuals often follow others’ opinions when forming their own [141].

**Figure 4:**
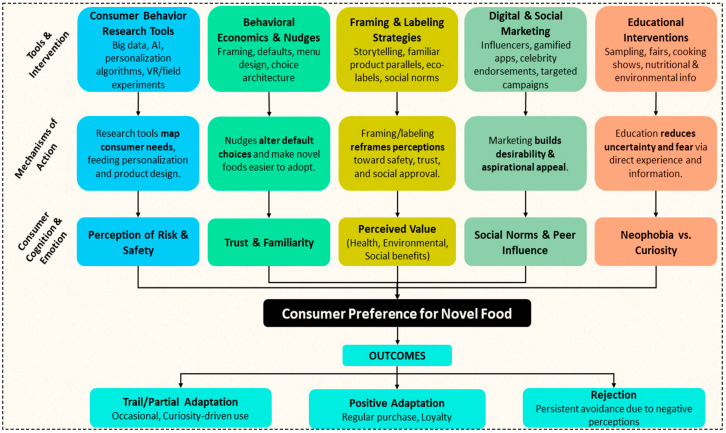
Mechanistic pathway showing interventions shaping consumer preferences for novel foods. The framework is based on a thematic synthesis of the literature and identifies key intervention domains (e.g., nudges, labeling, marketing, and education) that are best reported across studies. Consumer cognition (risk perception, trust, perceived value, social norms, and neophobia) is influenced by these interventions, which also affect preference and adoption outcomes (trial, sustained use, or rejection). The figure is a synthesis of the themes, rather than a quantitative model. To retain conceptual clarity and general applicability, highly product-specific, regionally constrained, or inconsistently reported factors were not factored in. The figure must therefore be understood as a conceptual synthesis of the major themes recognized in the literature, not as a comprehensive or quantitative model; as such, it is intended to summarize and integrate the key insights of this review.

Regardless, nudges are more strongly affected by their level of intrusiveness, and they are more effective when consumers believe their autonomy is valued, such as with straightforward opt-out options [142]. Emotional barriers, such as perceptions of unnaturalness and disgust, also affect consumer acceptance. Therefore, these barriers can be mitigated by enhancing trust and familiarity through educational campaigns and transparent labeling [57]. In short, thoughtfully planned nudging and behavioral economics approaches effectively address emotional responses and cognitive biases, thereby significantly improving consumer acceptance of novel foods. By integrating social norm cues, framing techniques, and default-setting steps to foster trust and familiarity, we can aid the shift toward more sustainable dietary alternatives. The conceptual framework of these interventions is presented in Figure 4.

### 4.3. Labeling and Framing Strategies

Labeling and framing methods influence consumer acceptance of plant-based alternatives and cultured meat. Chambers et al. [143] emphasized the significance of message framing in shaping consumer perspectives. Additionally, Lee and Lee [144] noted that messages emphasizing personal advantages, such as individual health and taste, elicited more optimistic responses than those highlighting societal advantages, including animal welfare and environmental conservation. It suggests that people prioritize direct personal benefits over societal benefits when considering new foods.

In labeling, visual cues significantly impact consumer perceptions; natural and realistic images of cultured meat enhance its acceptance by mitigating the perceived risks associated with its unfamiliar origin [144]. Conversely, abstract and technical imagery is often perceived as forcing skepticism. Images and technical descriptions, such as petri dishes, reduced acceptance of cultured meat; however, more relatable presentations and less technical language increased consumer perspectives [145].

Personal distinctions, such as sensation striving, also moderate these influences. Low-sensation aspirants responded positively to messages emphasizing personal advantages and direct food cues, showing that tailoring communication approaches can improve effectiveness [144]. Furthermore, researchers have noted that individuals’ motivations influence their responses to messages about novel foods. Sheng et al. [146] found that individuals who prioritize new opportunities and personal growth are more likely to appreciate messages highlighting the advantages of consuming artificial meat. In contrast, individuals who were more worried and cautious about avoiding risks favored messages emphasizing the risks of not consuming these novel foods.

In short, effective labeling and framing techniques that highlight individual benefits, employ natural and relatable imagery, and assess individual distinctions in consumer personality and motivation can significantly enhance consumer acceptance of novel foods. These strategies can reduce unfamiliarity and risks, encouraging more optimistic consumer attitudes regarding novel foods.

### 4.4. Educational Intervention

Educational interventions notably impact novel food acceptance by encouraging positive attitudes, mitigating food neophobia, and enhancing relevant knowledge through targeted approaches [147]. Educational drives, school-based programs, and workshops support the explanation of novel foods with scientific evidence of their health benefits and safety, thereby improving consumer willingness to try and consume them. The Nutrition Education-Culture of Wellness in Preschools in Early Childhood has revealed that interactive learning and repeated exposure can enhance the desire to consume or try novel foods, especially among ethnic minority and low-income groups [148]. Likewise, a 12-week Food Friends program initiative’s social marketing campaign has revealed promising outcomes in children’s eating behaviors, enhancing their willingness and preference to try novel foods [149].

Among teenagers, academic units focused on insect-based novel foods have effectively increased willingness to try and mitigate food neophobia [147]. Taste exposure and sensory education have also been promising in motivating 8- to 12-year-old children to consume novel and unfamiliar foods. These interventions typically employ repeated exposure, rewards, and peer modeling to enhance acceptance and decrease resistance [148].

Knowledge intervention also plays a vital role in helping adults accept upcycled and functional foods. A meta-analysis and systematic review revealed a positive relationship between consumers’ acceptance of novel foods and their level of knowledge, emphasizing the significance of increasing awareness through education [150]. Likewise, consumers with a better understanding of environmental issues and food waste are more likely to adopt upcycled foods made from food by-products. Effective communication regarding ethical considerations, sustainability, and health benefits is crucial to enhancing consumer acceptance [151]. Behavioral approaches integrated with education, such as the Smarter Lunchrooms Program, utilize simple strategies from behavioral economics and social psychology to encourage children to choose and try novel healthy foods. These interventions, such as visual cues and attractive naming, have helped improve the preference and consumption of nutritious foods [152].

Furthermore, social media and digital platforms are increasingly utilized to disseminate educational content, shaping awareness among young consumers about novel foods and their benefits. Educational interventions can increase consumer acceptance by reshaping perceptions, building trust, and improving knowledge. Contextually tailored, effectively designed interventions promote health-conscious, sustainable food alternatives while decreasing psychological barriers. For example, tasting-based interventions with primary school children in Belgium and the Netherlands have shown significant improvements in willingness to try insect-based foods. Programs combining nutritional information with cooking demonstrations among university students in Italy and Spain have increased acceptance of plant-based meat [153]. Target audience, country context, program duration, and delivery modality (e.g., classroom, online, and retail-based) are critical moderators of effectiveness that should be specified for each cited study.

## 5. Novel Food Preferences: Specific Case Studies

Various novel foods are gaining popularity due to consumer preferences. Recent case studies on insect proteins, precision fermentation, plant-based meats, cultured meat, and algae-based foods highlight both the challenges and the potential of these products to achieve widespread adoption (Table 2). In Table 2, studies were selected to represent breadth across novel food categories (plant-based meats, cultivated meat, insect proteins, precision fermentation products, and algae-based foods) and methodological approaches (surveys, experimental tastings, choice experiments, focus groups, and mixed methods). Priority was given to multi-country or cross-cultural studies and to studies with sample sizes exceeding 200 participants, as these offer greater generalizability. Studies are not claimed to be exhaustive; they are representative of the state of the literature within the 2020–2025 timeframe.

### 5.1. Plant-Based Meat Alternatives (PBMAs)

Nowadays, PBMAs have appeared as a primary novel food type driven by increasing consumer interest in ethical considerations, health, and sustainability. However, consumer adoption of PBMAs is mainly driven by perceived likeness to traditional meat, particularly in terms of texture and taste. In a cross-national study, Michel, Hartmann, and Siegrist (2021) found that ethical and environmental concerns increasingly influence consumers in the Netherlands and Germany, while texture and taste remain important factors in acceptance [156]. Likewise, Caputo, Sogari and Van Loo [139] performed a choice study integrated with sensory evaluations, demonstrating that meat’s overall likeness, flavor, and texture remain prominent in consumer reviews of PBMAs.

Sogari et al. [171] conducted a sensory study that identified the most and least liked sensory characteristics of PBMAs, demonstrating that mushy texture, unfamiliar or “off” flavors, and unpleasant aftertastes significantly discouraged repeated purchases. The sensory mismatch between experience and expectations is a primary factor in the general adoption of PBMAs, and improving textural fidelity and flavor calibration is necessary to enhance consumer acceptance. Additionally, a systematic review collected sensory evaluation data across PBMAs, supporting the notion that acquiring sensory satisfaction is key to consumer acceptance. Without meaningful progress in optimizing aroma, texture, and taste, PBMAs risk high trial-and-rejection rates, particularly among new adopters and omnivores. Therefore, sensory innovation remains a top priority for manufacturers seeking to transform curiosity into loyalty [41]. Swiss consumers considered 3D-printed and PBMAs to be environmentally friendly and nutritious, compared with insect-based or cultured proteins, thereby improving their willingness to consume repeatedly [172].

In PBMAs, after sensory attributes, price is often considered an essential element that influences consumer preference and decision making. A survey-based study of over 2000 participants in the USA demonstrated that price competitiveness is a more powerful factor than taste likeness in encouraging consumers to try PBMAs rather than traditional meat. However, health or environmentally conscious consumers look for affordability when making decisions [173]. Demographics and culture also significantly influence PBMA preferences. Consumers in Kuwait and Canada reported that personal beliefs and local norms, such as perceived environmental concerns, health benefits, and religious–cultural acceptance, significantly influenced their purchase intentions of PBMAs [174]. Likewise, Chia et al. [175] studied PBMA perception among multiethnic Asians and found notable divergence in consumer acceptance based on exposure to Western food, dietary background, and ethnicity.

Mental associations and social dynamics also play a significant role in shaping consumer mindsets toward PBMAs. White et al. [176] found, using Social Practice Theory, that PBMA consumption typically arises from routine-based practices shaped by peer influence, availability, and family habits, suggesting increased PBMA uptake through community-based campaigns and social channels. Similarly, USA consumers mentally classify PBMAs, revealing that many associated them with health and sustainability, while others still linked them with inferior taste, unfamiliarity, and artificiality. These mental links often override the rational advantages, so targeted marketing and positive framing are necessary for a behavioral shift [177]. In short, the appeal of PBMAs is growing as awareness of personal health and environmental sustainability. Successful consumer adoption relies on leading determinants, such as sensory satisfaction, while social norms, cultural identity, and pricing act as barriers to adoption. These case studies collectively highlight the multifaceted nature of consumer preferences and the significance of interdisciplinary approaches in promoting novel foods.

### 5.2. Precision Fermentation (PF) Products

Precision fermentation is a biotechnological technique to genetically modify microorganisms (such as fungi, bacteria, and yeasts) to produce fats and proteins traditionally obtained from plants and animals with minimal environmental impact. PF presents multiple potential advantages, including decreased agricultural land use, sustainable protein production, and a lower carbon footprint. Nevertheless, consumer acceptance of PF products is influenced by sensory characteristics, ethics, and various demographic factors [31]. An important PF product is Perfect Day’s dairy proteins (gray literature), which replicate animal-based proteins such as whey and casein to produce dairy-free alternatives, e.g., cheese, milk, and ice cream. Sustainability is a critical factor in consumer preferences, so Perfect Day products are attracting environmentally conscious consumers, particularly Gen Z and Millennials demographics who prioritize sustainability in their purchasing decisions [178].

Zollman Thomas, Chong, Leung, Fernandez and Ng [140] conducted a cross-cultural survey about PF-produced eggs in Singapore, the USA, and Germany to investigate consumer acceptance. The study revealed that higher-income groups had better acceptance than low-income groups. The outcomes underscored that the motivations for adopting this product differed: Singaporean consumers were driven by price and health concerns, Americans by health advantages such as cholesterol-lowering properties, and Germans by animal welfare concerns. Additionally, in Singapore, females were less willing to accept PF-produced eggs than males. In another study, Banovic and Grunert [179] examined the impact of framing on consumers’ perspectives regarding PF products and technologies. The results indicated that representing PF products as “natural” rather than “sustainable” evoked more optimistic attitudes towards them. Furthermore, highlighting the likeness of conventional fermentation and PF methods increased perceived benefits and trust, thereby growing consumer acceptance.

In Germany, a study examined consumer preferences for PF-produced cheese and found that animal welfare benefits and uniform product quality positively impacted consumer acceptance. Nevertheless, concerns about the potential economic effects on traditional farmers and the use of GMOs slightly reduced the willingness to try or consume PF cheese [180]. GlobalData (gray literature) revealed that plant-based dairy alternatives have gained popularity in Australia. However, concerns about cost, texture, and taste are hindering general adoption. Therefore, PF offers a solution by creating animal-free dairy alternatives that resemble conventional dairy items in appearance and taste.

Nevertheless, reluctance persists, with 66% of Australian consumers being unwilling to consume or try dairy substitutes, primarily due to concerns about texture and flavor [178]. Desire and curiosity to experience novel food alternatives can drive initial trials, even though continued acceptance may be based on repeated consumption and its benefits. It suggests that marketing plans emphasizing the distinctive qualities and environmental and health benefits of PF products could increase consumer adoption [140]. In short, consumer acceptance of PF products varied across ethical considerations, health consciousness, desire for novelty, and environmental awareness. Educational interventions that communicate the PF product’s safety, benefits, and quality are necessary for improving consumer acceptance. Addressing consumer concerns will be essential to their integration into mainstream markets [180].

### 5.3. Insect-Based Protein

Insect-based proteins are traditional foods that are novel to many contemporary consumers, and consumer preferences for them are shaped by sensory, psychological, cultural, and regulatory factors. Consumer acceptance of this practice is low across European societies despite its widespread presence in multiple parts of the world, particularly in the Democratic Republic of the Congo, Central African Republic, Zambia, Cameroon, Uganda, Zimbabwe, South Africa, and Nigeria [181]. The recent literature suggests that awareness of the nutritional benefits and sustainability of insect consumption is increasing despite low consumption rates. Recent research on Millennials and Gen Z has found that 86% of insects are recognized as nutritious, with 58% being associated with sustainability; however, only 18.6% of consumers have tried them. Additionally, participants (93.2%) found these products appealing when the natural insect appearance was concealed; nonetheless, conventional insect-free products still ranked higher in texture, sweetness, and taste. The persistence of this knowledge–behavior gap reflects the operation of multiple psychological mechanisms beyond an information deficit. High disgust sensitivity is one of the strongest predictors of insect food rejection, exhibiting low susceptibility to cognitive override through education alone [1]. Food neophobia and the absence of established social norms around insect consumption in Western contexts further inhibit translation of knowledge into trial. The effectiveness of sensory masking, while generating high initial trial rates, also raises questions about long-term acceptance: whether concealment of insect identity sustains repeat consumption or merely delays rejection upon disclosure is an underexplored area requiring longitudinal study [182].

Cultural perceptions, such as the “yuck factor,” limit consumer acceptance of insects in Western cultures, where insects are often viewed as pests rather than as food sources. In Africa, Latin America, and Asia, where entomophagy is formal, insects have better consumer acceptance. For instance, in Singapore, crickets are incorporated into conventional dishes following the approval of 16 insect species for human consumption. Traditional edible insect dishes consumed globally include: chapulines (toasted or fried grasshoppers seasoned with lime and chili, a staple of Oaxacan markets and tacos in Mexico); tamales de gusano de maguey (agave worm tamales, a pre-Columbian delicacy in central Mexico); fried mopane worms (Gonimbrasia belina, consumed with maize porridge in southern Africa); silkworm pupae stir-fried with garlic (beondegi in Korea); and grasshopper dishes prepared with herbs and spices in northern Thailand (to’ona). These examples illustrate that entomophagy is embedded in rich culinary traditions across three continents, offering culturally resonant reference points for novel food marketing [183]. A Hungarian case study found that 30% of consumers preferred edible insects, while 70% showed no preference. Among consumers of different ages and sexes, 27.6% of women aged 18–59 years showed mild interest in consuming edible insects [184].

Regulatory frameworks are also important; the European Union, under the EU Novel Food Regulation and the European Food Safety Authority (EFSA), has endorsed specific insect species for human consumption, aiming to ensure the sustainability and safety of insect-based products. Marketing techniques that highlight the nutritional value and environmental benefits of insect proteins while also addressing cultural and sensory concerns are crucial [182]. Reducing phobias and incorporating insect protein into everyday food have shown promising results. Insect powder, when incorporated into pasta, energy bars, and bread, can help mitigate insect visibility, thereby improving consumption. Results indicate that incorporation levels of 5–10% sustain good texture and taste [185]. Although insect-based proteins are a source of sustainable nutrition, consumer preference is influenced by improving sensory appeal, addressing cultural barriers, implementing effective marketing strategies, and navigating regulatory frameworks. Future research is crucial to bridging the gap between consumption and awareness, thereby encouraging the integration of insect protein into formal diets.

### 5.4. Cultured Meat

Over the last five years, researchers investigating consumer acceptance of cultured meat have made significant strides in identifying consistent cultural, sensory, and psychological determinants. Ethical concerns and knowledge of livestock-related carbon emissions have paved the way for sustainable alternatives such as cultured or lab-grown meat [186]. A systematic review combining 105 practical studies found that consumer acceptance of cultured meat is influenced by macro-level factors, including religion, culture, GDP per capita, meat consumption habits, population dynamics, and CO_2_ emissions; however, cross-national practical analysis remains limited [187].

Psychological barriers, including disgust sensitivity and food neophobia, are constantly recognized as the main hindrance to cultured meat consumer acceptance. A controlled study found that evoked disgust and perceived unnaturalness are significantly negatively associated with consumer willingness. These reactions trigger cognitive heuristics, especially the “natural-is-better” heuristic, which can deter the adaptation of cultured meat until it is properly addressed [54]. Emotional and novelty disgust are primary barriers to consumer preferences [187]. To et al. [188] reported that cultured meat’s promise is based on its capability to imitate conventional meat’s texture and flavor. Sensory attributes, such as mouthfeel, juiciness, tenderness, and taste, are crucial to consumer acceptance and satisfaction. Theoretical claims alone are inadequate without a convincingly expressed sensory trial.

A meta-review has identified the perceived advantages, including food security, animal welfare, and environmental sustainability, as the primary motivators for consumer consumption. However, concerns such as unnaturalness, food safety, and trust in science continually pose barriers [189]. Undoubtedly, results indicate that personal-level drivers, such as universalism and innovativeness, positively predict openness, whereas distrust, neophobia, and disgust in biotechnology act as internal barriers [187]. Furthermore, the marketing of cultured meat still faces notable barriers to consumer acceptance. Educational interventions significantly increased acceptance rates by up to 79% in controlled trials, indicating the potential of targeted communication interventions [186]. Rolland et al. [190] documented that 58% of consumers were willing to spend on cultured meat, suggesting less doubt among consumers and a greater likelihood of market acceptance. Therefore, a harmonized research plan that incorporates strategic framing, sensory science, cross-cultural considerations, and consumer psychology may help bring cultured meat to mainstream acceptance globally.

### 5.5. Algae-Based Foods

Consumer acceptance of algae-based foods is influenced by an intricate interplay among familiarity, nutritional expectations, sensory perceptions, and sustainability concerns related to the final product [191]. A cross-study involving the Netherlands, Spain, Germany, Italy, and Hungary, based on over 3000 participants, examined consumer willingness to try microalgae-based products, such as pasta, bread, plant-based sausages, and energy bars. Results revealed that the consumers ranked as “uninterested,” “uncertain,” and “enthusiast.” Factors affecting willingness to try included general health interest, naturalness, product pleasantness, animal friendliness, and sustainability. Particularly, consumers who recognized microalgae products as rich in protein, minerals, and vitamins were likely to be “enthusiasts” [192]. Microalgae can impart unique colors, odors, and flavors to food products, which could be unappealing or unfamiliar to most consumers. A study on breadsticks incorporating microalgae revealed that consumers initially anticipated significant variation, but their perceptions became more favorable after they tried them. Consumers associate breadsticks with health advantages and are also willing to pay a premium for them [22].

Nevertheless, challenges prevail in masking microalgae’s natural colors and flavors [193]. The incorporation of dairy products can cause unpleasant changes in taste, color, and texture, which may reduce consumer attraction [194]. To overcome these problems, microencapsulation has been studied to mask the odor and taste of microalgae, thereby improving sensory acceptance [193].

A Spanish-based study highlighted a lack of consumer knowledge about microalgae, particularly among the oldest and youngest populations. Regardless, microalgae were commonly known as safe, nutritious, and sustainable. The results of the study suggest that providing consumers with more information could improve the market share and acceptance of microalgae-enriched products [167]. Likewise, qualitative UK-based research has demonstrated that consumers lack sufficient knowledge about algae as a food source. Therefore, they were often willing to try algae-based products, particularly when incorporated into everyday food. Participants underscored the significance of product characteristics, including affordability, sustainability, and perceived health benefits. The integration of algae into familiar products and the transparent marketing of its environmental and health advantages were proposed to improve consumer acceptance [168]. Moreover, cooking oils derived from algae offer an appealing option for health-conscious consumers, with high smoke points and neutral taste while being environmentally conscious. They are praised for their sustainability and versatility, while higher costs hinder adoption. In short, managing sensory concerns through technological innovations, transparent marketing, and enhancement of consumer cognition can encourage the integration and acceptance of algae into daily diets [168].

## 6. Limitations of This Review

This review has several limitations that should be considered when interpreting its findings. First, the restriction to the literature published between January 2020 and August 2025 means that foundational pre-2020 studies, including seminal work establishing the food neophobia scale [195], the theory of planned behavior [196], and early consumer research on GM foods, are incorporated only selectively for theoretical grounding. Readers seeking a comprehensive historical overview are directed to earlier systematic reviews [197]. Second, the narrative synthesis methodology, while appropriate for integrating studies with heterogeneous study designs, is inherently more susceptible to selection bias than a fully systematic meta-analytic approach. However, a structured database search and explicit inclusion/exclusion criteria were applied. The final synthesis involved qualitative judgment in selecting and weighing individual studies, which may introduce confirmation bias despite the authors’ efforts to balance them.

Third, the included literature exhibits a pronounced geographic skew toward Western, high-income populations, particularly Northern and Western Europe, North America, and Australia. Consumer populations in South and Southeast Asia, Sub-Saharan Africa, and Latin America, where novel food acceptance may be substantially higher for some categories (e.g., insect proteins) and lower for others (e.g., cultured meat), are underrepresented. Generalizations from this review to non-Western contexts should therefore be made cautiously. Fourth, gray literature (industry reports, regulatory documents, and market research) was selectively included where no peer-reviewed equivalent was available. Such sources may carry commercial interests that influence the framing of consumer acceptance data. Where gray literature is cited, the source type is identified, and findings are triangulated with peer-reviewed evidence where possible. Fifth, the rapid pace of development in novel food technology and regulation means that some findings, particularly regarding cultivated meat regulatory approvals, precision fermentation commercialization, and AI-driven marketing practices, may have been superseded by developments occurring after the August 2025 search date. Future reviews should update this synthesis as the evidence base matures.

### Synthesis of Evidence and Strength of Findings

Drawing together the evidence reviewed in preceding sections, several higher-order insights emerge that transcend individual food categories and methodological domains. These are organized by evidence strength and consistency across studies. High-consistency findings (supported by convergent evidence across multiple study designs and cultural contexts): First, sensory experience, particularly taste and texture, consistently emerges as the primary determinant of initial trial and repeat purchase across all novel food categories. This finding holds across surveys, experimental tasting, and neurophysiological methodologies, making it the most robust generalizable conclusion of this review. Second, food neophobia significantly moderates acceptance, with high neophobia predicting rejection of novel foods independently of product category, culture, or study design. Third, younger age, higher education, and environmental concern are consistently positive predictors of novel food acceptance, though effect sizes vary considerably across studies and should not be overstated.

Moderate-consistency findings (supported by multiple studies but with meaningful heterogeneity): First, health benefit framing increases acceptance in some consumer segments but triggers processing-related skepticism in others, particularly among food-literate consumers who scrutinize ingredient lists. The direction of this effect is moderated by product category and health claim specificity. Second, transparency and traceability labeling generally increase trust. However, its impact depends on the specific production method disclosed: “precision fermentation” labels elicit more positive responses than “genetic modification” labels for functionally equivalent products, reflecting the power of terminology framing over objective content. Third, social norms and peer influence effects are consistently identified as important in survey-based studies. However, experimental studies demonstrate that these effects are substantially weaker in actual purchase contexts than self-reported data suggest, a recurring attitude–behavior gap in the literature.

Low-consistency or contradictory findings requiring interpretive caution: First, the relationship between environmental concern and willingness to pay for sustainable novel foods is inconsistent across studies; several report no significant premiums among self-identified environmentalists, suggesting a well-documented attitude–behavior gap. Second, the effectiveness of disgust habituation interventions (e.g., insect food tastings) is mixed: some studies report lasting reductions in neophobia post-exposure, while others find that disgust rebounds to baseline within weeks. Third, AI personalization and digital nudging effects on novel food purchase behavior remain understudied with small, predominantly Western samples, limiting generalizability. These graduated assessments of evidence strength are intended to guide practitioners and policymakers in prioritizing intervention levers and to signal to researchers where additional high-quality cross-cultural and longitudinal evidence is most urgently needed. To operationalize the synthesis, evidence was categorized into three tiers using predefined criteria applied uniformly across all included studies (Table 3).

## 7. Research Opportunities with Future Directions

**Figure 5:**
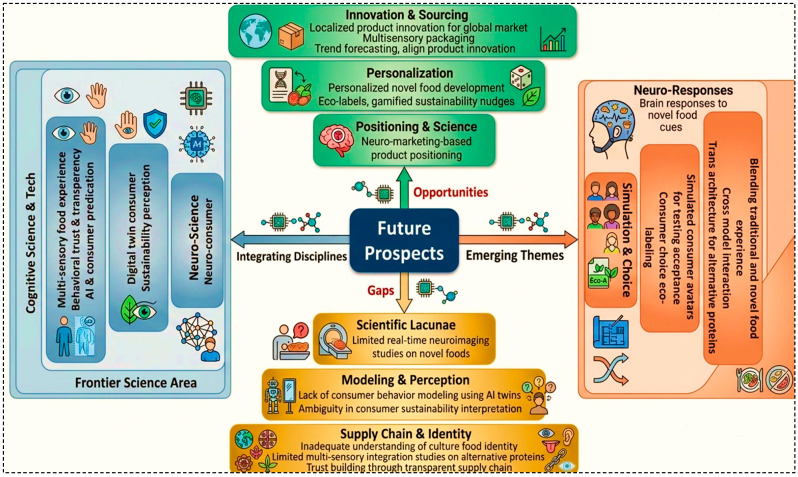
Future research areas, emerging gaps, and recommendations for directing consumer behavior toward purchasing and consuming novel foods.

Future studies on novel food preferences should prioritize cross-cultural and long-term analyses, as recent findings are often confined to short-term perspectives and Western surroundings. Exploring how evolving values, cultural norms, and exposure influence consumer acceptance in the long term will enable us to understand various consumer behaviors, especially those associated with less familiar products, such as algae and insects (Figure 5). Combining cutting-edge sensory and emotional analytics, including neurogastronomy and biometric feedback, can enhance our understanding of consumer self-reported food choices. In novel food acceptance, neurogastronomy examines how brain–sensory interactions affect flavor perception, satiety, and the overall eating experience, while biometric feedback can capture real-time emotional and physiological responses during tasting. Both provide deeper insights into the underlying mechanisms that shape consumer acceptance of novel foods. These approaches may provide accurate emotional feedback on appearance, texture, and taste, which can affect whether a product is accepted or rejected.

AI offers promising ways to deliver personalized and segmented food recommendations to consumers. Future research should investigate how to integrate food preferences, behavioral traits, and health data using machine learning approaches to develop personalized dietary recommendations, thereby enhancing the acceptance and relevance of novel foods. In addition, labeling and framing strategies require further investigation. Although terms such as “eco-friendly” and “natural” have shown positive effects, more evidence is needed to understand how these cues interact with consumer perception of trust and naturalness. Cross-cultural and demographic studies on communication framing could help optimize acceptance strategies. Furthermore, research should examine how peer norms, online trends, and influencer marketing shape consumer perceptions, as well as how digital ecosystems may either reduce or intensify skepticism towards novel foods.

Psychological, cultural, and ethical frameworks should be combined to adequately address food disgust, environmental values, and moral identity. Real-world (supermarket or app) behavioral tests can assess interventions such as eco-labeling, sensory sampling, and default options to measure accurate consumer decisions. Stakeholders should invest in AI-based marketing and sensory optimization approaches to enhance their marketing plans and overall impact. Academic researchers should encourage interdisciplinary collaboration, and policymakers should support consistent regulation, public education, and transparent labeling to build trust. NGOs should promote cultural inclusivity and accessibility by adopting fair pricing models and engaging in community meetings. In prioritizing future research efforts, it is valuable to distinguish the highest-leverage gaps from secondary areas. The most critical priorities include: (1) longitudinal studies tracking conversion from initial trial to sustained dietary incorporation; (2) cross-cultural validation of constructs (e.g., disgust sensitivity and neophobia) using harmonized instruments; (3) equity research on how socioeconomic factors modulate adoption across settings; and (4) mechanistic studies linking sensory–emotional responses to repurchase behavior. Prioritizing these would move the field toward causal, predictive, and actionable knowledge.

## 8. Conclusions

This review presents an integrated conceptual framework that maps cross-domain interactions of behavioral economics, sensory science, food policy, and digital marketing, moving beyond parallel domain summaries toward a more coherent analytical model. It also provides explicit operational definitions for key terminology, which may help reduce ambiguity in cross-study comparisons. A graduated assessment of evidence strength is offered, distinguishing high-consistency findings that can inform immediate policy and practice from contradictory or understudied findings that require further investigation. This review also acknowledges the geographic, methodological, and temporal limitations of the evidence base, providing a more targeted future research design. The findings indicate that consumer interest in novel foods is driven primarily by perceived health benefits, ethical considerations, and environmental sustainability. However, significant barriers remain, particularly high product pricing, cultural resistance, and low sensory acceptability. These challenges are not uniform across populations or contexts, suggesting that tailored approaches, such as consumer education, digital marketing campaigns, and evidence-based framing interventions, may be more effective than one-size-fits-all strategies. For practice, this implies that product development should prioritize sensory quality and cost competitiveness alongside sustainability messaging. For policy, targeted communication strategies that address specific consumer concerns in different markets are recommended. Future research should prioritize longitudinal studies on consumer behavior, cross-cultural comparative analyses, and intervention effectiveness trials to strengthen the evidence base. Addressing these gaps will be essential to facilitating the broader adoption of novel foods and their potential contribution to global food system sustainability.

## Figures and Tables

**Table 2:** Globals perceptions of willingness to try/purchase novel foods, along with driving factors and barriers.

Product Type	Research Location	Sample Size	Approach	Willing to Try/Purchase	Driving Factors	Barriers to Consumption	Ref.
Plant-based meat alternative	Austria (Germans)	433	Choice experiment	Not numeric; part-worth utilities estimated	Price preferences, origin, and ingredient	Ingredient unfamiliarity and price sensitivity	[154]
	Australia	1078	Cross-sectional online survey	Consumers’ willingness to try plant-based meat alternatives included semi-vegetarians/flexitarians (19.6%), full-time vegetarians (4.2%), and vegans (0.6%).	Environmental friendliness, animal welfare, health, safety, affordability, and sensory factors	Being less affordable and enjoyable	[28]
	Australian	679	Online survey promoted via social media	74% of respondents had tried at least once in their life; moreover, the majority were willing to try to purchase in the future	Vegetarian nature, ethics, and flexitarians (actively reducing meat intake)	Health concerns and lack of trust in claims	[155]
	UK	2000	Survey	Try: 73%; purchase: 59%	Sustainability and healthiness	Lack of familiarity and taste	[156]
	Germany	500	Quantitative online survey	47.4% had tried or wanted to purchase or consume hybrid meat in the future	Health, sustainability, and animal welfare	Inability to fully mimic real meat taste	[157]
	USA	Over 1800	Discrete choice experiment (DCE)	Sixteen percent of males and females were willing to purchase or try the product	Novel food, low cost-effectiveness, vegetarian nature, and nutritional value	Being non-vegetarian	[158]
	USA	1800+	DCE	The percentage of respondents willing to buy, consume, or try was 7%	Pocket friendliness, vegetarian nature, and nutrition	Being non-vegetarian	[158]
Lab-grown or cultured meat	Netherlands and Finland	376 (126 from the Netherlands and 250 from Finland)	Online survey (Qualtrics)	65% in the Netherlands and 63% in Finland	Country, diet, age, gender, animal welfare, and environmental friendliness	Food neophobia and unawareness of environmental friendliness	[159]
	Netherlands and Finland	376 (126 + 250)	An online survey (via Qualtrics)	72 and 73.2% in the Netherlands and Finland, respectively	Country, diet consciousness, age, gender, familiarity, and knowledge of food sustainability	Food neophobia and nutrition unawareness	[159]
	Netherlands and Finland	376–126 for the Netherlands and 250 for Finland	Online questionnaire application (Qualtrics)	66.2 and 65.6% in the Netherlands and Finland, respectively	Country, age, gender, and food sustainability knowledge	Fear of trying new foods	[159]
	Australia	1078	Cross-sectional online survey	After receiving information about lab-grown meat, one-quarter of respondents were willing to eat lab-grown chicken or beef	Young age, education, positive beliefs regarding eating experience, animal welfare, familiarity, and healthiness	Health, affordability, environmental friendliness, eating enjoyment, and safety	[28]
	USA and UK	2292 from the US and 2270 from the UK	Online survey	80% of the target population in both countries were willing to try cultivated meat, 40% were moderately likely to, and 40% were highly likely to	Product familiarity, provision of supporting narratives, attractive labeling, a willingness to try innovative food, and a desire to purchase genetically engineered food	Unfamiliarity, trying labeling, novel food neophobia, and low-likeness perception towards GMOs	[160]
	USA	Over 1800	DCE	5%	Appealing nutrition and animal welfare	Labeling as “beef”	[158]
Insect-based protein/powder/burger	13 countries	~8100	Online survey (CATA)	Significantly differ by country, format, and insect type	Novelty and protein sustainability	Less acceptance in whole-insect formats, and differ by culture	[161]
	Gabon and Belgium	821 (412 + 409)	WTP measurement + KAP questionnaire	High willingness to try; Gabonians not willing to pay a premium but Belgians willing to	Novelty, price appropriateness, familiarity, and health perception	WTP constraints and cultural acceptance (lower in Gabon)	[162]
	Poland (Gen Z)	1063	Online CAWI survey	13% approved processed insects in veg dishes; 9% had consumed them before	Ecological reasons (flexitarians and pescatarians)	Vegetarian ethics and unfamiliarity	[163]
	Finland	1000	Online questionnaire	In total, 24% of the respondents expressed intention to increase their future consumption of insect-based protein products	Healthiness and sustainability	Disgust, food neophobia, and affordability	[164]
	China, USA, France, UK, New Zealand, Netherlands, Brazil, Spain, and the Dominican Republic	3091	Survey (quantitative research design)	Willingness to try: 23.2% Willingness to purchase: 16.9% Willingness to pay more: 7%	Nutritional benefits, suitability, and healthiness	Food neophobia, sensory attributes, and environmental impacts	[165]
Plant-based protein	Finland	1000	Online survey (questionnaire)	26% of respondents planned to increase their use of insect-based protein products	Healthiness, sustainability motives, and reduced fear of trying novel foods	Taste and nutrition	[164]
	Finland	600	Mixed methods	Purchase: 44%; try: 58%	Local innovation and lactose-free	Cultural habits and taste	[166]
	China, US, France, UK, New Zealand, Netherlands, Brazil, Spain, and the Dominican Republic	3091	Quantitative survey	Willingness to try: 40.8% Willingness to purchase: 34.8% Willingness to pay more: 17.5%	Suitability, protein benefits, general importance of food healthiness, and sustainability	Dietary factors, nutritional concerns, and taste	[165]
	12 countries (incl. UK, USA, etc.)	4488	Omnibus online survey 2018–19	Willingness for plant-based protein ~20% ≈ moderate	Nutrition, health, sustainability, and safety	Texture/taste vital, underscored as a key inhibitor	[165]
Precision-fermented egg	Singapore/USA/Germany	3006	Likert for purchase/try	Try: GER 61%, USA 51%, and SGP 56% Purchase: GER 57%, USA 48%, and SGP 48% Regular: GER 34%, USA 30%, and SGP 26%	SGP: price and health; US: curiosity and health; Germany: animal welfare	Varied by country, education, income, novelty concerns, and DNA mentions	[140]
Microalgae product/meat substitutes	Spain	85	Real-restaurant sensory test	Majority positive acceptance	Open to a slight price premium and healthy perception	Color/flavor/odor differences were detectable but did not deter acceptance	[22]
	Spain	3084	Questionnaire survey	0.1% consumed regularly, and 8% had tried occasionally; positive intention if informed	Environmental friendliness, sustainability, and health	No habit of use and lack of information/knowledge	[167]
	UK	34	Focus groups	No % given, general openness to try	Novelty, affordability, health, edibility, and sustainability	Unfamiliarity with algae as a protein alternative and limited knowledge	[168]
	Singapore	Unspecified	Survey	Positive willingness is associated with health and sustainability	Health and sustainability	Unfamiliar flavors and lower taste expectations	[169]
	Germany, Belgium, Sweden, and Italy	413	Sensory evaluation (liking+ CATA)	Liking the highest for *blue Spirulina* and *Lithothamnium calcareum*	Familiarity with crackers vs. neophobia, familiar sensory profiles (“sweet,” “toasted bread”)	Speckled appearance and off-flavors (“umami,” “fishy”)	[170]

**Table 3:** Criteria and examples for evidence strength classification.

Evidence Strength	Criteria for Assignment	Representative Examples from the Review
High Consistency	Convergent findings in ≥3 studies ≥2 study designs (e.g., survey + experimental + neurophysiological) Replication in ≥2 cultural/geographic contexts ≤20% of studies report contradictory effects	Sensory experience (taste/texture) as primary driver of trial and repeat purchase. Food neophobia as a consistent moderator of novel food acceptance. Younger age, higher education, and environmental concern as positive predictors.
Moderate/Mixed Consistency	Supported by ≥2 studies Meaningful heterogeneity in effect direction/size Predominantly single-method evidence (e.g., self-report surveys without behavioral validation) OR a well-documented attitude–behavior gap present	Health benefit framing increases acceptance in some segments but triggers skepticism in food-literate consumers. Transparency labeling increases trust, but the effect depends on terminology (“precision fermentation” vs. “genetic modification”). Social norms are important in surveys but weaker in actual purchase contexts.
Limited/Contradictory Evidence	Supported by <3 studies >30% of studies show conflicting/opposing results Small sample sizes (<50/group) Single-country (predominantly Western) samples No longitudinal follow-up for interventions	Environmental concern and willingness to pay for sustainable novel foods (inconsistent premium). Disgust habituation interventions (e.g., insect tastings)—mixed lasting effects vs. rebound to baseline. AI personalization and digital nudging effects—understudied, small Western samples.

## Data Availability

No new data were created or analyzed in this study. Data sharing is not applicable to this article.
